# A novel Gerstmann-Sträussler-Scheinker disease mutation defines a precursor for amyloidogenic 8 kDa PrP fragments and reveals N-terminal structural changes shared by other GSS alleles

**DOI:** 10.1371/journal.ppat.1006826

**Published:** 2018-01-16

**Authors:** Robert C. C. Mercer, Nathalie Daude, Lyudmyla Dorosh, Ze-Lin Fu, Charles E. Mays, Hristina Gapeshina, Serene L. Wohlgemuth, Claudia Y. Acevedo-Morantes, Jing Yang, Neil R. Cashman, Michael B. Coulthart, Dawn M. Pearson, Jeffrey T. Joseph, Holger Wille, Jiri G. Safar, Gerard H. Jansen, Maria Stepanova, Brian D. Sykes, David Westaway

**Affiliations:** 1 Centre for Prions and Protein Folding Diseases, University of Alberta, Edmonton, Alberta, Canada; 2 Department of Medicine (Neurology), University of Alberta, Edmonton, Alberta, Canada; 3 National Research Council of Canada, Edmonton, Alberta, Canada; 4 Department of Electrical and Computer Engineering, University of Alberta, Edmonton, Alberta, Canada; 5 Department of Biochemistry, University of Alberta, Edmonton, Alberta, Canada; 6 Brain Research Centre, University of British Columbia, Vancouver, British Columbia, Canada; 7 Canadian Creutzfeldt-Jakob Disease Surveillance System, Centre for Foodborne, Environmental and Zoonotic Infectious Diseases, Public Health Agency of Canada, Ottawa, Ontario, Canada; 8 Department of Clinical Neurosciences, University of Calgary, Calgary, Alberta, Canada; 9 Hotchkiss Brain Institute and Calgary Laboratory Services, University of Calgary, Calgary, Alberta, Canada; 10 Departments of Pathology and Neurology, School of Medicine Case Western Reserve University, Cleveland, Ohio, United States of America; 11 Division of Anatomical Pathology, University of Ottawa, Ottawa, Ontario, Canada; Istituto Superiore di Sanità, ITALY

## Abstract

To explore pathogenesis in a young Gerstmann-Sträussler-Scheinker Disease (GSS) patient, the corresponding mutation, an eight-residue duplication in the hydrophobic region (HR), was inserted into the wild type mouse PrP gene. Transgenic (Tg) mouse lines expressing this mutation (Tg.HRdup) developed spontaneous neurologic syndromes and brain extracts hastened disease in low-expressor Tg.HRdup mice, suggesting *de novo* formation of prions. While Tg.HRdup mice exhibited spongiform change, PrP aggregates and the anticipated GSS hallmark of a proteinase K (PK)-resistant 8 kDa fragment deriving from the center of PrP, the LGGLGGYV insertion also imparted alterations in PrP's unstructured N-terminus, resulting in a 16 kDa species following thermolysin exposure. This species comprises a plausible precursor to the 8 kDa PK-resistant fragment and its detection in adolescent Tg.HRdup mice suggests that an early start to accumulation could account for early disease of the index case. A 16 kDa thermolysin-resistant signature was also found in GSS patients with P102L, A117V, H187R and F198S alleles and has coordinates similar to GSS stop codon mutations. Our data suggest a novel shared pathway of GSS pathogenesis that is fundamentally distinct from that producing structural alterations in the C-terminus of PrP, as observed in other prion diseases such as Creutzfeldt-Jakob Disease and scrapie.

## Author summary

Prion diseases can be sporadic, infectious or genetic. The central event of all prion diseases is the structural conversion of the cellular prion protein (PrP^C^) to its disease associated conformer, PrP^Sc^. Gerstmann-Sträussler-Scheinker Disease (GSS) is a genetic prion disease presenting as a multi-systemic neurological syndrome. A novel mutation, an eight amino acid insertion, was discovered in a young GSS patient. We created transgenic mice expressing this mutation and found that they recapitulate key features of the disease; namely PrP deposition in the brain and a low molecular weight proteinase K (PK) resistant internal PrP fragment. While structural investigations did not reveal a gross alteration in the conformation of this mutant PrP, the insertion lying at the boundary of the globular domain causes alterations in the unstructured amino terminal portion of the protein such that it becomes resistant to digestion by the enzyme thermolysin. We demonstrate by kinetic analysis and sequential digestion that this novel thermolysin resistant species is a precursor to the pathognomonic PK resistant fragment. Analysis of samples from other GSS patients revealed this same signature, suggesting a common molecular pathway.

## Introduction

Gerstmann-Sträussler-Scheinker Disease (GSS) is an autosomal dominant multi-systemic neurological syndrome that may evolve to frank dementia and often exhibits a protracted clinical course [1]. This inherited amyloidosis is caused by a number of mutations in the human gene, *PRNP*, on chromosome 20 that encodes the cellular prion protein (PrP^C^) [2]. PrP^C^ is a GPI-linked protein displayed on the cell surface; its N-terminus is natively unstructured and contains tandem metal binding octarepeats and a hydrophobic region (HR). PrP^C^'s C-terminal region folds into a globular three-helix bundle with a small two-stranded β sheet [3]. In infectious prion diseases, PrP^C^ refolds to a pathogenesis-associated form, PrP^Sc^, whose structure is dominated by β sheet [4–6].

GSS is of interest as there is a propensity to form amyloid, sometimes accompanied by formation of neurofibrillary tangles (NFTs) [7, 8] and because there is only a limited capacity for the generation of infectious titre and/or ability to transmit to recipient species [9]—these properties thus offering a partial parallel to Alzheimer's Disease, where Aβ amyloid and NFTs are pathologic hallmarks in idiopathic and genetic disease [10] and where transmissibility of clinical disease to animal recipients is not a hallmark [11]. With poor GSS transmission first shown for experiments using non-human primates [12], transmissions into non-Tg rodents are similarly inefficient and stand in contrast to Creutzfeldt-Jakob Disease (CJD) [13]. This inefficiency may be partly overcome by using mice engineered to match the donor PrP allele [14, 15], or by using bank voles [16], which are promiscuous hosts for a number of prion diseases [17–21]. Clinical presentation of GSS can be variable and partially dependent on the causative mutation in the *PRNP* gene coding region, the most common clinical phenotypes are cerebellar ataxia and pyramidal signs with eventual cognitive decline before death [22]. Multicentric plaques composed of truncated PrP fragments are often found in the brains of these patients and spongiosis may or may not be present [23].

A notable *PRNP* mutation was recently discovered in a young GSS patient. The index case presented with status epilepticus at age 34, prefaced by night terrors at age 26. While the parasomnias subsided after a 6-year period, memory problems and behavioural changes emerged at this age [24]. *PRNP* sequencing revealed homozygosity for valine at the polymorphic residue 129 and heterozygosity for a partial internal duplication, resulting in a protein with 8 extra residues. This LGGLGGYV insertion lies at the junction between the HR—the most conserved area of PrP and one reported to adopt alternative membrane topologies that are associated with neurodegeneration [25–28]—and the globular domain, where it is predicted to duplicate some of the residues found in the first β-strand of PrP (residues 128–131 [29]). Prompted by findings that early structural rearrangements in the PrP^C^ to PrP^Sc^ transition involve the HR [30] and the possibility of discovering a process underlying an aggressive form of GSS disease, we pursued animal modeling. Our studies define a novel misfolded form of mutant PrP^C^ that prefigures the 8 kDa PrP fragment pathognomonic for end-stage GSS with multicentric amyloid plaques [31] and may be shared by other allelic forms of GSS; they also suggest an explanation for the early disease onset of the index case.

## Results

### Spontaneous and transmissible prion disease in transgenic HRdup PrP mice

**Fig 1:**
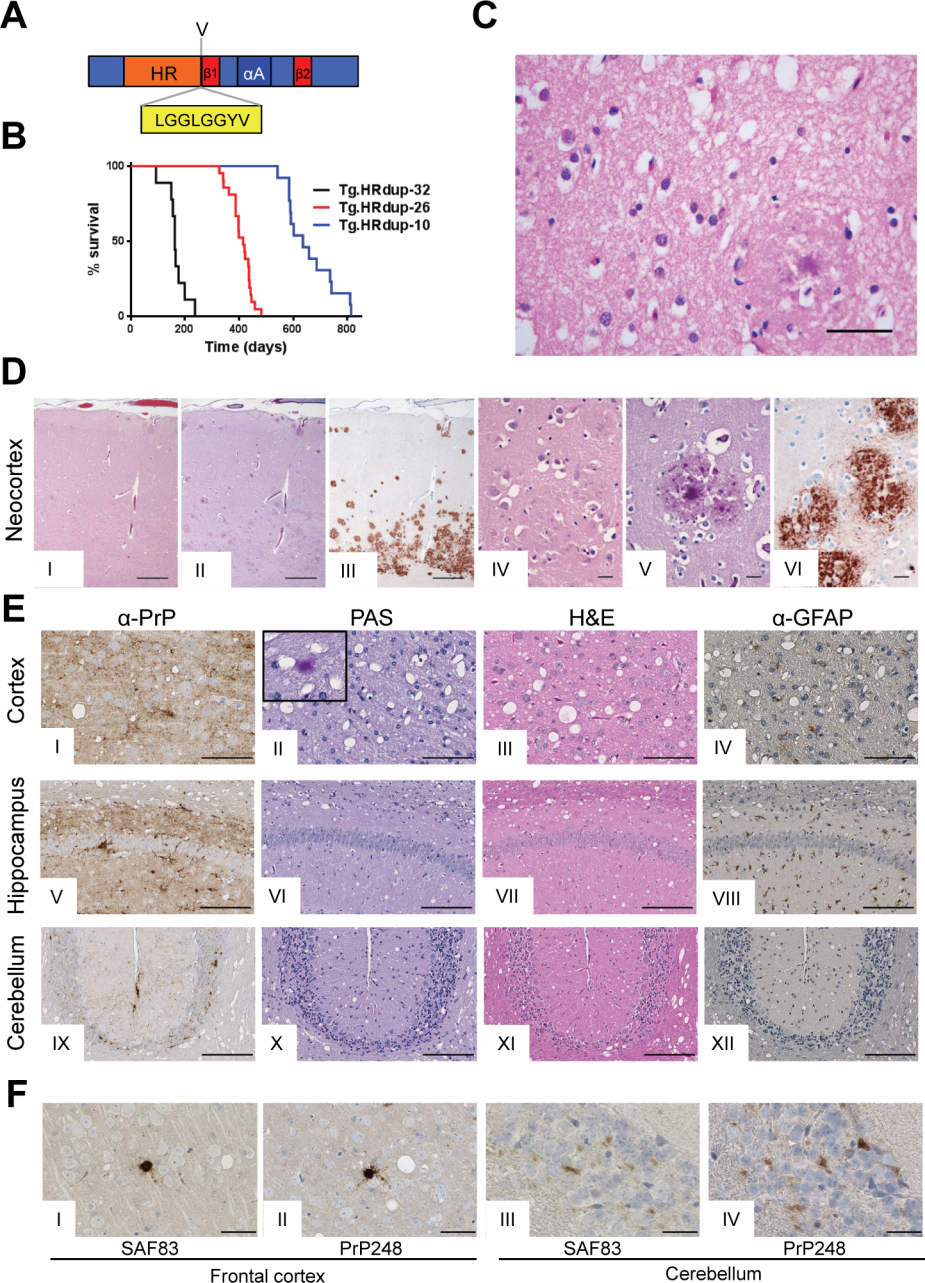
HRdup PrP causes GSS in transgenic mice. A) Line diagram of PrP demonstrating the site of the 8 amino acid insertion (yellow box) in the center of PrP. The hydrophobic region, "HR", is shown in orange, β-strands (strand 1 and strand 2, "S1" and "S2") are in red and helix A in dark blue. The presence of valine at res. 128 is indicated. B) Kaplan-Meier survival plot of the transgenic lines expressing HRdup PrP. Tg.HRdup-32, 166 ± 39 days (black, SD; n = 9); Tg.HRdup-26, 410 ± 40 days (red, SD; n = 23) and Tg.HRdup-10, 662 ± 76 days (blue, SD; n = 16). Panels C & D, Pathological features of the index GSS patient. C) Focal neocortical moderate spongiosis (middle frontal gyrus), not preferentially associated with MCP. H&E staining, scale bar = 50 μm. D) Low power views of the cortex in H&E, PAS and 12F10 antibody stain (I-III) and a higher power view (IV-VI) with size bars of 500 μm and 25 μm, respectively. E) Pathological features of Tg animals with spontaneous disease. Photomicrographs of sagittal brain sections of Tg.HRdup-26 mice at terminal stage of disease (panels I-XII). Immunostaining for PrP was performed after treatment of the slices with formic acid while slices for examination with H&E (third column) or GFAP (fourth column) were left untreated. The CA1 region of the hippocampus is shown. Inset in panel II shows a PAS-positive plaque in the cortex. Scale bar = 25 μm. F) High power view of cortical (I and II) and cerebellar (III and IV) PrP deposits in Tg.HRdup mice stained with two antibodies, as indicated. Scale bar = 50 μm.

In analyses of RK13 cells lacking endogenous PrP^C^ we concluded that glycosylation, endoproteolysis, biotinylation of cell-surface PrP and immunocytochemistry with and without cell permeabilization was similar between WT and HRdup and M128V allelic variants of mouse PrP (S1 Fig). We generated three lines of Tg mice expressing mouse PrP with the 8-residue insertion in the HR between amino acids V128/L129 (mouse numbering scheme, Fig 1A, Table 1), Tg.Prnp.HRdup.M128V-32, Tg.Prnp.HRdup.M128V-26 and Tg.Prnp.HRdup.M128V-10 (for brevity, Tg.HRdup-32, Tg.HRdup-26 and Tg.HRdup-10). We also created two control lines expressing a mouse *Prnp* allele modified to incorporate the equivalent of the human valine 129 polymorphism, Tg.Prnp.M128V-39 and Tg.Prnp.M128V-25 (Tg.M128V-39 and Tg.M128V-25). Since GSS alleles are dominant in their natural setting, transgenes were expressed on an FVB/N *Prnp*^+/+^ genetic background. The Tg lines differed in their net steady-state levels of PrP^C^, with expression levels ranging from 3.4–1.6x endogenous for the mutant lines and 1.9–2.0x for the control M128V lines (Table 1). With aging, Tg.HRdup mice, but not Tg.M128V mice, developed a spontaneous neurologic syndrome with accompanying neuropathological changes wherein animals with the highest transgene expression levels succumbed to the syndrome faster than their lower expressing counterparts (Tg.HRdup-32 > Tg.HRdup-26 > Tg.HRdup-10; Table 1, Fig 1, S2 and S3 Figs); while the life expectancy of Tg.HRdup-10 mice was not significantly shorter than that of non-Tg FVB/N littermates in our colony, neurological presentation and a discrete pathological signature (below) distinguished it from age-related death.

**Table 1:** Expression level, disease onset and signature PrP fragments in transgenic mice.

Parameter	Tg.HRdup-32	Tg.HRdup-26	Tg.HRdup-10	Tg.128V-39	Tg.128V-25
Net expression level relative to WT (1x)	3.4x	2.6x	1.6x	2.0x	1.9x
Age euthanized (days)	166 ± 39	410 ± 40	662 ± 76	spontaneous neurologic disease not detected	spontaneous neurologic disease not detected
8 kDa PK-resistant fragment in diseased mice^a^	not detectable	yes	intermittent	N/A	N/A
16 kDa thermolysin- resistant species in adolescent mice^a^	ND	yes (7 days)	yes (15 days)	ND	ND
16 kDa thermolysin- resistant species in aged mice^a^	yes	yes	yes	not detectable	ND
Age euthanized post inoculation^b^	N/A	N/A	270 ± 38	N/A	N/A

ND, not done; N/A, not applicable.

^a^ analysis of brain homogenate without PTA precipitation.

^b^ inoculated with brain material from clinical phase Tg.HRdup-26 mice. *Tga20* mice inoculated with the same material were held for an observation period of 400 days with no signs of clinical disease.

The most notable clinical feature of Tg.HRdup mice was slowly progressing ataxia. As the disease advanced, weight loss was apparent and the animals were euthanized when showing kyphosis and hypokinesia. Tg.M128V mice with comparable PrP expression levels did not present with any neurologic abnormalities and were used throughout the study as negative controls (Table 1). Since myopathy has been described in GSS mice expressing the P102L allele (P101L in mouse PrP [32]), we sought these pathological changes in Tg.HRdup-26 animals with clinical disease; these studies failed to define necrotizing myopathy or neuropathy (S4 Fig), suggesting that these types of lesions do not contribute to the clinical presentation of Tg.HRdup mice.

As attempts to transmit GSS isolates to primate and rodent models have yielded varied successes [16, 22, 33] we explored the issue of transmissibility using Tg.HRdup-10 animals as recipients for brain homogenate from sick Tg.HRdup-26 animals and observed acceleration of disease course from 662 ± 76 to 270 ±38 days post-inoculation (Table 1). Tg.HRdup-10 animals inoculated with non-transgenic, healthy brain homogenate culled at 399 days post inoculation had no signs of disease (Table 1, S5 Fig) while *tga20* mice (which express WT PrP^C^ at ~6-7x endogenous levels and thus higher than the other Tg lines in our study [34]) remained healthy and displayed no pathology when inoculated with the same brain homogenates from Tg.HRdup-26 animals in the clinical phase of disease. These data demonstrate that pathogenic processes in the brains of the Tg.HRdup mice extend to the generation of infectivity and offer a parallel to prior studies of transmission/host-range effects when a GSS P102L mutation is inserted into a mouse *Prnp* gene [15].

### Histopathology in the index case and induced by HRdup PrP transgenes

Previously reported neuropathological data on the index case were restricted to a right frontal lobe biopsy. The formalin-fixed tissue obtained at autopsy showed only mild to focal moderate spongiosis, with mild gliosis in the neocortex and cerebellum molecular layer (Fig 1C; Fig 1D I and IV); other areas showed no significant spongiosis. Vacuolation and gliosis of the hippocampus, cortex and cerebellum were present in all three Tg.HRdup lines but these features were most prominent in Tg.HRdup-26 animals (Fig 1E).

The index case showed most intense multicentric plaque (MCP) burden, as visualized with α-PrP antibodies and Periodic Acid-Schiff (PAS) stain, in the neocortex and slightly less in the cerebellar molecular layer and hippocampus. Moreover, in the neocortex there was a layer oriented distribution of MPCs. Layer 5 and 6 showed most immunostaining with aggregates of MCPs (Fig 1D; II, III, V and VI), followed by layer 1, and the least MCPs in layer 2 and 3. The cerebellum showed less MCPs at the arachnoidal side of the molecular layer and most at the Purkinje cell side. There was no association between plaques and vacuoles (Fig 1D; I and III). Somewhat less intense PrP deposits were found in other grey matter structures including striatum and thalamus with the most sparse and smallest deposits present in the brainstem with granular synaptic deposits in inferior olivary nucleus and dentate nucleus.

Tg.HRdup-26 animals showed a similar distribution of PrP plaques, with other affected areas being the anterior olfactory nucleus, corpus callosum, thalamus, anterior commissure and medulla. Some focal PrP deposits in mice stained with PAS (Fig 1E; II, inset). PrP deposits stained with monoclonal antibodies for C-terminal and N-terminal residues are shown in Fig 1F. In the case of Tg.HRdup-10 animals, the most intense PrP deposition was found in the cerebellum (S2 Fig) whereas, interestingly, analogous deposits were scarce in Tg.HRdup-32 animals which succumbed to disease ~400 days earlier. These data are summarized in S1 Table. Aged-matched Tg.M128V-39 animals were negative for all these histopathological hallmarks (S3 Fig). In the case of disease produced by inoculation of Tg.HRdup-10 mice with Tg.HRdup-26 brain extracts, there was an accentuation of spongiform change and focal PrP deposition in the cerebellum (S5B Fig) versus changes seen in aged un-inoculated Tg.HRdup-10 mice (S2 Fig).

In the index case, Aβ, phosphorylated tau and proteasome-targeted proteins or inclusions were not detected. In agreement with this, attempts with AT8 antibody (phospho Ser202, phospho Thr205) to detect hyper-phosphorylated tau in Tg mice with spontaneous disease were unsuccessful.

### Proteinase-K resistant signature fragments in HRdup PrP transgenic mice

**Fig 2:**
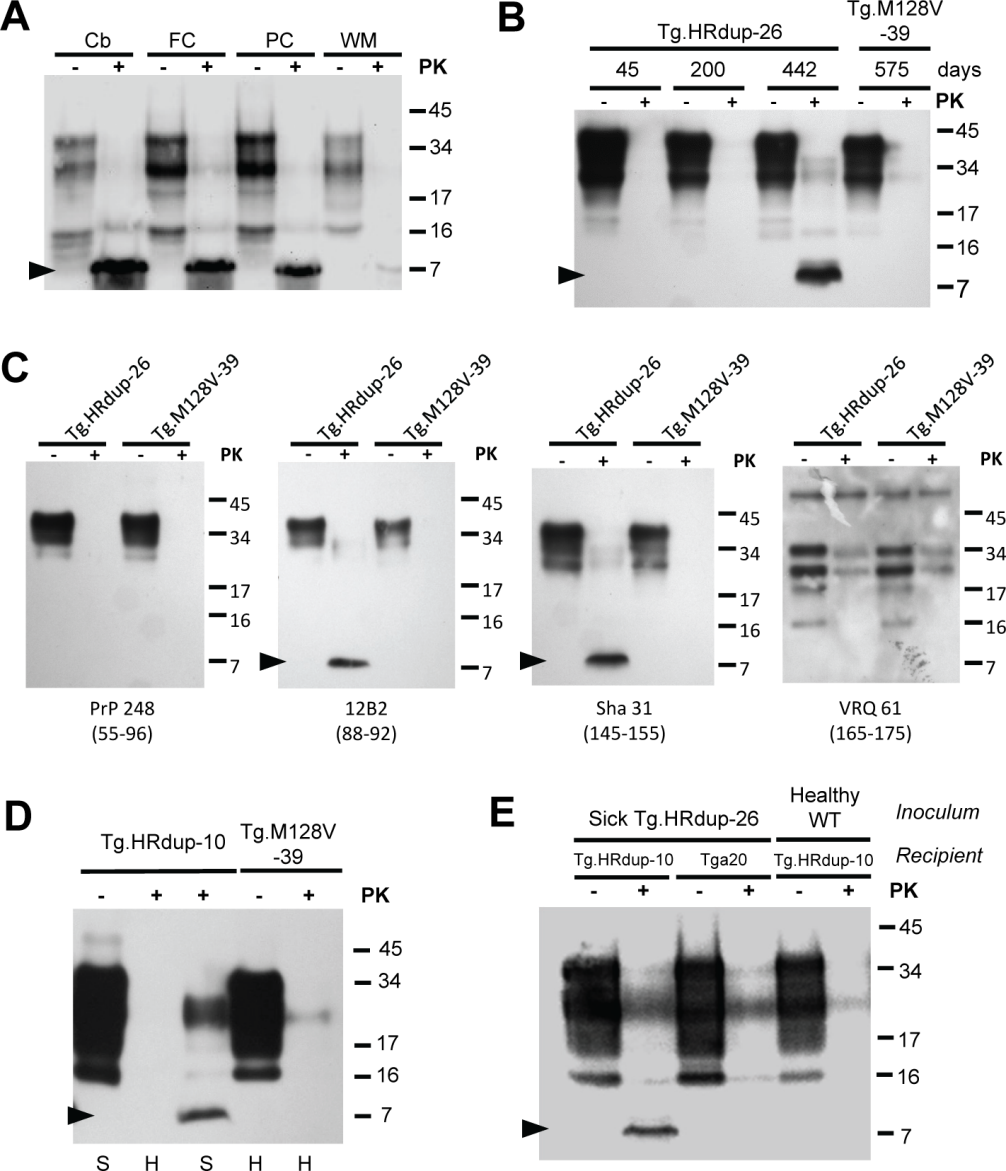
PK-resistant PrP in the index case, spontaneously sick and inoculated Tg mice. A) Presence of proteinase K (PK)-resistant PrP in different brain regions of the index case. Cb, cerebellum; FC, frontal cortex; PC, parietal cortex; WM, white matter. Black arrow indicates the position of the 8 kDa PrP fragment. 250 μg of protein was digested with 10 μg/ml of PK in a volume of 250 μl. B) Age-dependent accumulation of the 8 kDa fragment in Tg.HRdup-26 mice but not in Tg.M128V-39 control animals. 250 μg of protein was digested with 10 μg/ml of PK in a volume of 250 μl. C) Immunoreactivity of the 8 kDa fragment versus four α-PrP antibodies. PrP248 recognizes the octarepeat region (residues 55–96) of PrP and fails to detect the 8 kDa fragment. 12B2 (residues 88–92) is reactive with the 8 kDa fragment. Sha31 (residues 145–152) recognizes the 8 kDa fragment. VRQ61 recognizes the β2-α2 loop (residues 165–175) of PrP but is not immunoreactive with the 8 kDa fragment. Using these epitopes as minimal termini, the fragment maps to residues 88–155 using the numbering scheme of WT mouse PrP, which corresponds in turn to a molecular weight of ~7.2 kDa. D) Presence of protease-resistant PrP in Tg.HRdup-10 mice but not in aged Tg.M128V-39 mice. "H", healthy"; "S", sick. At the terminal stage of disease, HRdup prions are resistant to digestion by 10 μg/ml PK. E) Presence of the 8 kDa fragment in low-expresser Tg.HRdup-10 mice is hastened by inoculum with brain homogenate from a Tg.HRdup-26 mouse. Low expressing Tg.HRdup-10 animals and *tga20* (overexpressing WT PrP ~6x) animals received an intracranial inoculation with brain homogenate from a sick Tg.HRdup-26 animal. Tg.HRdup-10 animals inoculated with healthy brain homogenate were euthanized after 400 days. Tg.HRdup-10 animals inoculated with brain homogenate from a sick Tg.HRdup-26 animal succumbed to disease at 276±38 days. Brain homogenates from inoculated animals either undigested or exposed to PK is presented. Note that only Tg.HRdup-10 animals inoculated with Tg.HRdup-26 brain homogenate display the 8 kDa PK resistant fragment (black arrow) versus genotype-matched controls or animals treated with control inoculum (right four lanes). Analyses in A, B, D and E were performed with Sha31 antibody.

Proteinase K (PK)-resistant fragments from the center of PrP and of a molecular mass estimated to be between 6–8 kDa distinguish GSS from the C-terminal fragments that accumulate in CJD [35–38]. For simplicity, we will refer to this fragment in the context of the index case and Tg mice as being "8 kDa" (but also noting that its molecular mass is predicted as 0.735 kDa greater than a PK-resistant fragment generated from the corresponding WT sequence). We investigated this situation using frozen brain tissue obtained at autopsy from the index case (Fig 2A). The deep white matter had a paucity of neuropathological change and comprised an internal control for these analyses; notably, the abundance of the 8 kDa fragment normalized for protein loading was correlated with neuropathology visualized by light microscopy. This 8 kDa signature was abundantly present in aged Tg.HRdup-26 mice with spontaneous disease (Fig 2B and 2C (middle panels)) and present in brain extracts from Tg.HRdup-10 mice with spontaneous disease (Fig 2D), but not in Tg.HRdup-32 mice (Table 1). It was absent from aged Tg.M128V-39 control mice (Fig 2B, 2C and 2D). Antibody mapping confirmed the 8 kDa species originates from the center of the PrP molecule, with 12B2 (res. 88–92) and Sha31 (res. 145–155) epitopes present, but with PrP248 (res. 55–96) and VRQ61 (res. 165–175) epitopes absent (Fig 2C); thus the fragment minimally consists of residues 88–155, in broad agreement with studies of a GSS-associated 7 kDa amyloid extracted fragments mapped to residues ~90–153 [38]. In terms of spontaneous pathogenic processes, the 8 kDa signature fragment was absent in young (healthy) Tg.HRdup-26 mice but was observed in animals with neurological symptoms of disease (Fig 2B). With respect to pathogenesis produced by inoculation, a paucity of this fragment in young, un-inoculated Tg.HRdup-10 mice was overcome in age-matched animals administered brain samples from sick Tg.HRdup-26 mice, whereas the same inoculum administered to over-expresser *tga20* mice did not yield the signature fragment (Fig 2E). Regarding the ability to propagate the 8 kDa fragment, these data are in agreement with GSS inoculations performed using prion-susceptible bank voles and Tg mice [16].

### Assembly of the HRdup PrP into high molecular weight complexes

**Fig 3:**
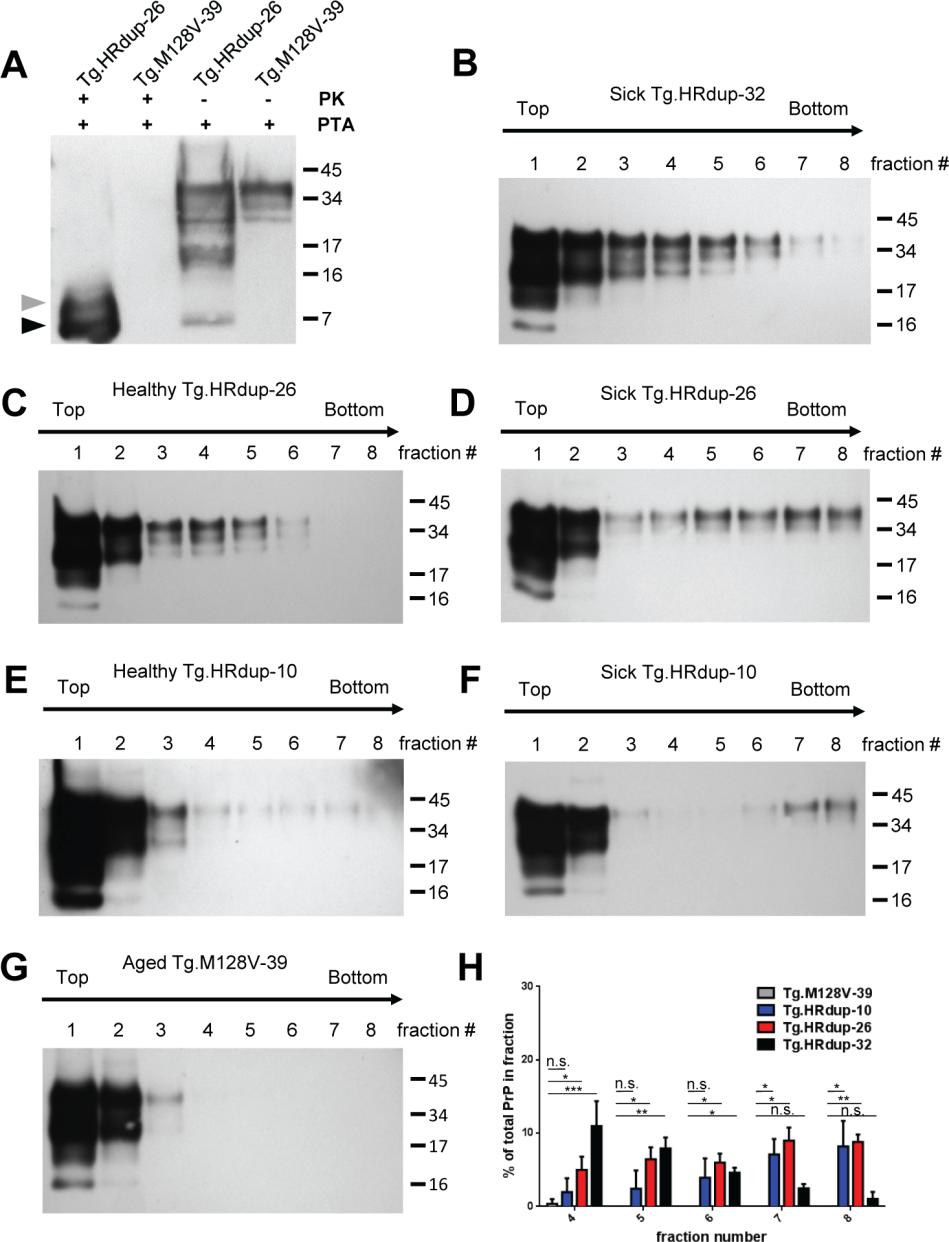
*In vivo* and *in vitro* properties of HRdup prions. A) The 8 kDa PK-resistant fragment is formed *in vivo*. Following PTA precipitation, the 8 kDa fragment can be observed by western blot without the use of PK (indicated by black arrow and a sub-molar species with a grey arrow), while it is absent from control mice (Tg.M128V-39). B-G) Sedimentation properties. PrP in both sick and healthy Tg.HRdup animals is found in lower fractions of linear 10–45% sucrose gradients in 1% Sarkosyl than in aged Tg.M128V animals. PrP immunoreactivity emerges in the lowest fraction at end-stage of disease, when PrP becomes PK-resistant while, in asymptomatic mice, PrP remains in the upper fractions of these gradients. B) A sick Tg.HRdup-32 animal. C) A healthy Tg.HRdup-26 animal. D) A sick Tg.HRdup-26 animal. E) A healthy Tg.HRdup-10 animal. F) A sick Tg.HRdup-10 animal G) A healthy aged Tg.M128V-39 animal. The Sha31 antibody was used for all blots. H) Quantification of PrP in fractions 4–8 from B, D, F and G. n = 3; statistics done using a one-sided students t-test; n.s., not significant; *, p< 0.05; **, p< .005; ***, p< .0005.

In samples from aged human brain, the action of endogenous proteases upon misfolded PrP (but not WT PrP^C^) can generate a natural protease-resistant domain. As phosphotungstic acid (PTA) precipitation [39] has been used previously to enrich for abnormal PrP in brain homogenates from P101L mice [40], we applied this procedure to process brain samples from our Tg mice. Subsequent western blot analysis of PrP revealed an 8 kDa signature in sick Tg.HRdup-26 mice, but not in control Tg.M128V-39 mice (Fig 3A). Immunoreactive species migrating between the positions of the 16 and 17 kDa molecular weight size marker were also noted. These data suggest a process wherein misfolded PrP assembles into higher molecular weight aggregates such that a core structure is not digested to completion by endogenous proteolytic processes (Fig 3A). To assess the quaternary state of PrP, we performed ultracentrifugation assays utilizing a linear gradient of 10–45% sucrose in the presence of 1% Sarkosyl; we analyzed brain homogenates from three biological replicates for each allelic type. These velocity centrifugation studies (Fig 3B–3G) revealed that HRdup PrP populates higher molecular weight gradient fractions (highest fraction numbers) in comparison to M128V PrP. Comparing sick Tg animals to their healthy genotypic counterparts (Fig 3C *versus* 3D, 3E *versus* 3F) revealed more signal in high molecular weight fractions such as fractions 7 and 8 whereas signal was not detected at all in fraction 4–8 of Tg.M128V animals (Fig 3G). Sick Tg.HRdup-32 mice showed a profile similar to healthy Tg.HRdup-26 mice, which is in line with the absence of PK-resistant PrP in these animals (Fig 3B *versus* Fig 3C). These analyses suggest that, in the context of the HRdup mutation, the development of PK resistant PrP detected by immunoblotting and focal PrP aggregates revealed by immunostaining is associated with detergent insoluble aggregates that are present in the bottom fractions of the gradient (summarized in Fig 3H).

### Stability and β-sheet structure of HRdup PrP

**Fig 4:**
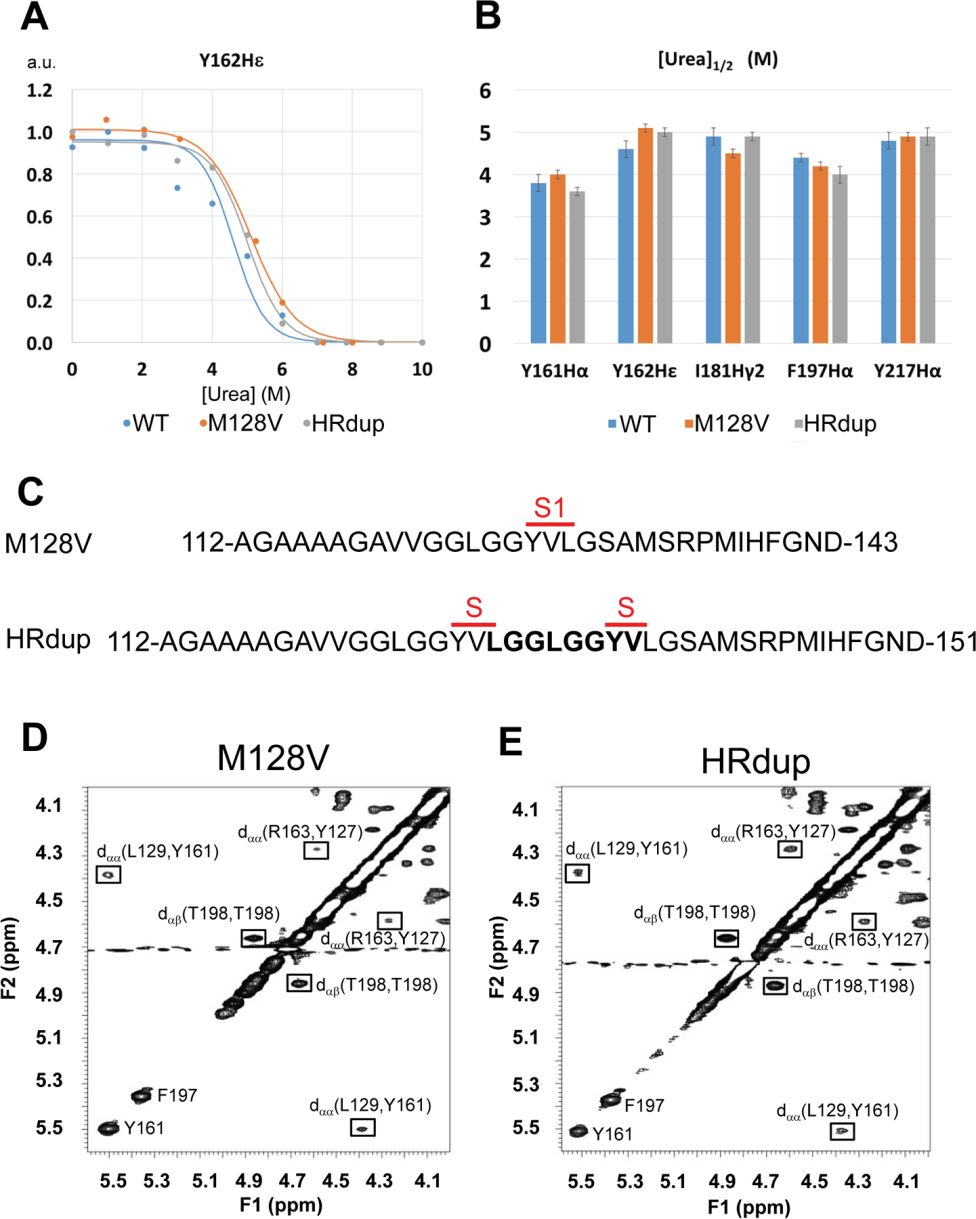
NMR spectra and beta-sheet signatures of recombinant proteins. A) Representative urea titration of one (Y162) of five assayed residues positioned in PrP's globular domain, as assessed for three PrP constructs (as indicated). B) Half maximal urea concentrations of each of the five residues with allelic origin color code as per panel A and presented with residues in numerical order. No pair-wise comparisons between the same residue measured in the three alleles reached significance. C) Position of beta strand S1 in WT PrP (top line) and a potential, additional beta strand, both indicated by S, that might arise as a consequence of the tandem duplication encompassing the residues YVLG. Lower panels; Part of the 2D ^1^H-^1^H NOESY spectra of M128V (D) and HRdup (E) where NOEs due to the presence of β-sheet are displayed.

We next turned to secondary and tertiary structure. Recombinant PrPs corresponding to WT, M128V (residues 118–231) and HRdup (residues 118–231 with an extra eight amino acids) were expressed by standard procedures. ^1^H NMR spectroscopy revealed all proteins were highly enriched and folded into a primarily α-helical structure (S6 Fig). To evaluate the stability of HRdup PrP, we performed a urea denaturation series (Fig 4A and 4B). Using previous chemical shift assignments of WT PrP, five resonances were identified in protons of the following residues: isoleucine181, Hγ2; phenylalanine197, Hα; tyrosine161, Hα; tyrosine217, Hδ; and tyrosine162, Hε. Stacked 1D spectra of samples under different urea concentrations are shown in S7 Fig. As previously performed to determine the stability of hamster, mouse, rabbit and bovine PrP [41], peak area values as a function of added urea were normalized to the largest value during denaturation process and then plotted against urea concentration. Two thermodynamic parameters, [D]_1/2_, the urea concentration at half point of unfolding and *m*, the slope of the denaturation curve which reflects the sensitivity of each resonance towards urea were extracted. Denaturing curves and [D]_1/2_ or *m* half values (S2 Table) for all five chosen resonances reveal that HRdup PrP had a similar sensitivity towards urea denaturation as WT and M128V PrP. Next, since the insertion in the HRdup allele includes extra residues as found within β-strand 1 in WT PrP, 2D NMR experiments were used to assess the short β-sheet structure in HRdup PrP. Here two characteristics indicate the presence of β-sheet structure: the downfield shift of Hα resonances [42] and presence of nuclear Overhauser effects (NOEs) within the α-H region (4 ppm to 5 ppm), due to the short distance between α-Hs from opposing residues in β-sheets. Within the α-H region of both HRdup and M128V we observed the NOEs from the two spectra as having the same chemical shifts, which indicate identical β-sheet structures formed by the same residues in the two sequences (Fig 4C–4E, S8 Fig). These NOEs are also consistent with those reported for the same region of WT mouse PrP [29]; this spectral information allowed assignments the NOEs in our spectra leading to the conclusion that the eight extra amino acids do not disrupt the short β-sheet structure present in all reported mammalian PrP NMR structures. However, these analyses are not able to determine if β-strand 1 of HRdup is composed of residues 127–130 (Tyr, Val, Leu and Gly) or residues 7–8 of the insertion and residues 129–130, or a mixture of the two.

### Molecular dynamics simulations of M128V and HRdup PrP

**Fig 5:**
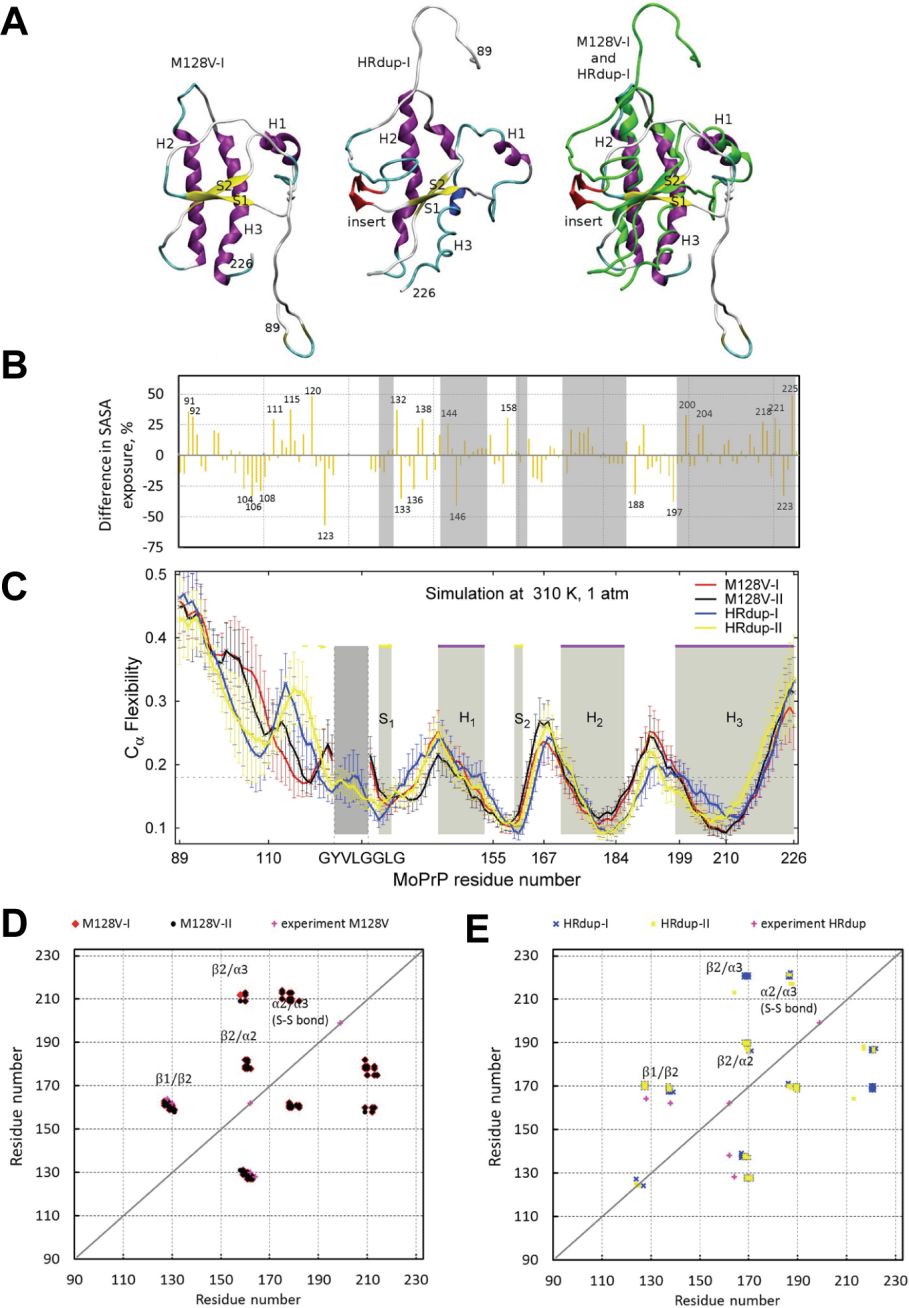
Molecular dynamics assessment of HRdup PrP. A) Representative conformations from 20ns molecular dynamics runs for M128V and HRdup PrP. α-helices are colored with magenta, β-strands with yellow, turns with cyan, random coils are white and the insert (for HRdup) is red. For the alignment, regions in HRdup that show differences with respect to M128V are depicted in green, and the insert is indicated in red. B) Differences in weighted average per-residue SASA for HRdup versus M128V models were averaged over two trajectories per allele (positive or negative differences indicate, respectively, greater or lesser solvent exposure in HRdup in comparison with M128V, as listed in the last column of S5 Table). Positions of helices and beta strands are shaded in grey (see also panel C). C) Main chain flexibility profiles in the PrP systems. For M128V, the flexibility profile is shown with red and black lines, and for systems with insert the profiles are shown with yellow and blue lines. The insert area is marked with vertical dashed lines. The main secondary structure elements in PrP are shaded in grey are indicated at the top of the plots with purple lines for α-helices and yellow lines for β-strands. Panels D) and E): The strongest correlations of non-consecutive residues from ECD pair correlation maps of the four PrP systems, as listed in S5 Table. D) Data for M128V-I (red dots) and M128V-II (black dots); E) Data for HRdup-I (blue crosses) and HRdup-II (yellow crosses). The regions of strongest correlations S1-S2, S2-H2, S2-H3, and H2-H3 are indicated. Correlations S1-S2 determined from 2D NOESY experiments are shown with magenta crosses in D and E.

Further analyses used molecular dynamics (MD) to probe PrP structure. Two MD trajectories for molecules with GYVLGGLG inserted between G125 and G126, the preferred model identified by homology modeling, ("HRdup-I" and "HRdup-II") and two for molecules without the insert ("M128V-I" and "M128V-II") were simulated at a temperature 310 K and pH 4.5 for 20 ns each, with secondary structure (SS) elements shown in Fig 5A and S9 Fig (see also S3 Table). Both M128V structures retained all major secondary structure elements including β-strands S1 and S2 and three α-helices H1, H2, and H3, with the exception of a small C-terminal part of H2 (Fig 5A and S9A and S9B Fig). In system HRdup-I, the N-terminus of helix H2 and almost the entire helix H3 unfolded (Fig 5A and S9C Fig). Residues insG6 and insL7 replace G123, and L124, forming a new beta strand. In system HRdup-II (S9D Fig) helix H2 and the middle part of helix H3 unfolded. Transient β-content was occasionally observed in N-terminal area of M128V-I, and to a greater extent in the areas of the insert and loop S2-H2 in HRdup-I and HRdup-II (S4 Table). To quantify structural differences between the alleles we averaged the per-residue solvent-accessible areas (SASA) [43] over the last 2 ns from the four MD trajectories (Fig 5B and S5 and S6 Tables). Two trajectories for HRdup and those for M128V exhibit close total SASAs for various groups of residues, as well as for the entire protein (S5 Table), and average per-residue SASAs were also close in HRdup and in M128V. However, the insert caused local effects (Fig 5B). Changes in SASA were evident in extended regions such as P104-K109, V111-V120, V121-G125, A132-D146, P164-Q167, N173-V179, V188-M204. (Fig 5B and S5 Table). Only a few residues with charged side-chains exhibited large differences in SASA suggesting a dominating effect on the hydrophobic residues.

Next, PrP dynamics were analyzed by an essential collective dynamics tool (ECD). ECD results supported the aforementioned trends, with main-chain flexibility profiles [44–47] of the PrP constructs shown in Fig 5C. High levels of the flexibility descriptor represent loops, whereas minima indicate rigid areas such as α-helices or β-strands. In both constructs the areas of helices H2 and H3 are characterized by broad minima indicating a relative rigidity whereas helix H1 is less stable, which is not unusual for PrP [44, 45]. The insertion caused extended regions of increased rigidity N-terminal to the insert and in the area of L108, where a systematic decrease in SASA was also observed (Fig 5B). The insert also seemed to destabilize the N-terminal part of helices H2 and H3 (Fig 5C) while increasing SASA in the same area (Fig 5B). Overall, data in Fig 5B and 5C indicates that an increase in per-residue SASA is often associated with greater main-chain flexibility, and *vice versa*.

ECD pair correlation maps (S10 Fig) provide complementary information on dynamic correlations of atomic motion within the same statistical-mechanical framework [46, 47], with S7 Table listing non-consecutive residues that show the strongest dynamic correlations in this type of analysis; Fig 5D and 5E graph these data and compares them with the 2D NOESY experiments. In summary, for M128V, the strongest correlations were found between β-strands S1 and S2, between β-strand S2 and helix H2, between helices H2 and H3, and between β-strand S2 and helix H3. For HRdup, the correlated elements are similar but the number of strongly correlated residues in H2 and H3 was notably decreased, whereas an additional correlation was observed between residues insG8 and Y127 with N-terminal residue A116.

Lastly, given the role of protein assembly effects in prion biology, protein/protein docking was assessed using representative trajectories (S11 Fig and S8 Table). Beyond intermolecular contacts found in top dimer model based on M128V-I and M128V-II, new contacts appeared or became more frequent in the N-terminus (W98, K100, A114, A115) and helix H3 (Y225, D226) of the HRdup-I and HRdup-II constructs. N-terminal contacts involving W98, K100, V111, and A116 were also frequently observed in heterodimers (S8 Table).

### A thermolysin-resistant PrP signature in GSS

**Fig 6:**
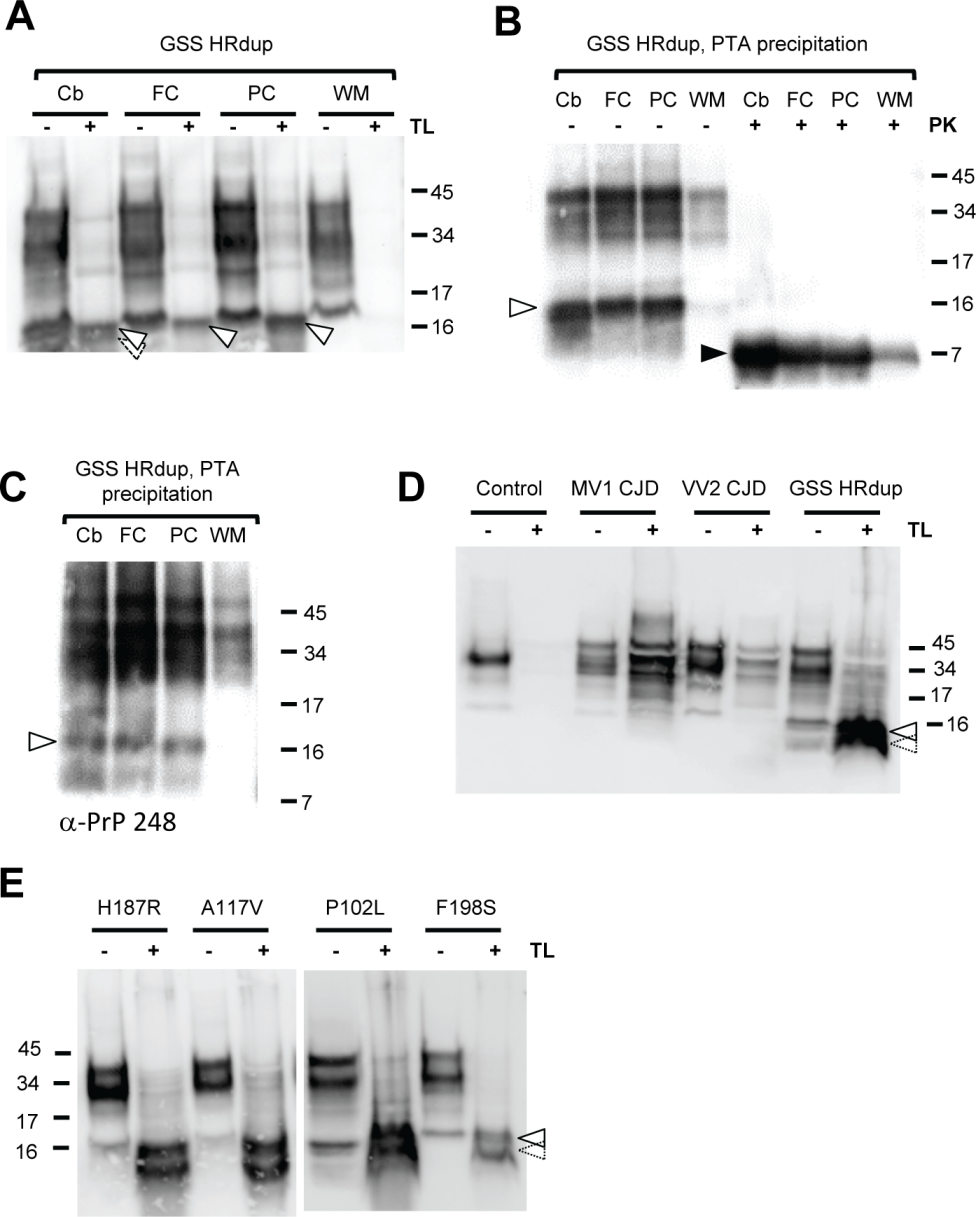
A 16 kDa thermolysin-resistant PrP signature in human brain material. A) Exposure of homogenates from different brain regions of the HRdup GSS patient to thermolysin. Cb, cerebellum; FC, frontal cortex; PC, parietal cortex; WM, white matter. Arrows indicate the position of the 16 kDa PrP species. 50 μg of protein was electrophoresed, either left undigested or exposed to thermolysin. B) PTA precipitates of lysates identify a prominent 16 kDa species (open arrow) and an 8 kDa species after PK digestion (black arrow), Sha31 antibody. C) PTA precipitates analyzed with PrP248 antibody (octarepeat epitope) reveals a 16 kDa species in Cb, FC, and PC. D) Thermolysin digests of MV1 and VV2 forms of sporadic CJD samples alongside a normal brain and an HRdup control. 50 μg of protein was exposed to thermolysin while undigested lanes correspond to 10 (control) or 5 μg total protein. E) Thermolysin digests of brain material from other forms of GSS; with the exception of P102L, the presented mutations are in *cis* to a valine codon at residue 129. 50 μg of protein was exposed to thermolysin while undigested lanes correspond to 10 μg total protein. Heterogeneity in the 16 kDa species is indicated by arrows with solid or dashed perimeters; see also Fig 7B. Analyses in A, B, D and E were performed with Sha31 antibody.

The protease thermolysin was used as an enzymatic probe of conformation to interrogate PrP^C^'s natively disordered region [48, 49]. Thermolysin will completely degrade PrP^C^ (see below) but produces a signature that includes protease-resistant full-length PrP^Sc^ from diseases such as mouse-adapted scrapie, hence providing information about the accessibility of residues in the natively disordered N-terminal region. For samples taken at autopsy, digestion of normalized protein samples from the index case from neuroanatomical areas with notable pathology (cerebellum (Cb); frontal cortex (FC); parietal cortex, PC)) yielded an intense 16 kDa thermolysin resistant species (Fig 6A) appearing as a doublet in the cerebellar sample, while the 16 kDa signal from an area with less pathological staining (deep white matter (WM)) was less notable. A 16 kDa species was also present in PTA precipitations from the same four tissue samples processed without any *in vitro* protease digestion (Fig 6B, Sha 31 antibody, lanes1-4). Here the relative abundance Cb ~FC ~PC > WM was again noted. While an 8 kDa species was not apparent in these analyses of the PTA precipitates of human material (Fig 6B, lanes 1–4), the same samples did yield an 8 kDa PrP fragment after PK digestion (Fig 6B, lanes 5–8) with a similar profile of signal intensity as noted above with thermolysin digestion, namely three robust signals (Cb ~FC, ~PC) versus a smaller signal (WM). A 16 kDa species was also seen with an octarepeat antibody in undigested PTA-precipitates (Fig 6C). Next, to address disease specificity, we sought similar signatures in sporadic CJD material or in normal brain. Two types of sCJD case containing a V129 polymorphism did not yield strong 16 kDa species but instead multiple species closer to the mobility of undigested PrP (Fig 6D). A normal control did not produce any TL-resistant species (Fig 6D) while cerebellar material from the proband yielded a doublet running slightly slower than the 16 kDa marker. To assess generality and the possibility that the 16 kDa signature can co-exist with or prefigure the generally accepted appearance of 7–8 kDa PK resistant species present in different types of GSS [37, 50, 51], we analyzed other GSS cases; these were pre-selected from samples within the US CJD surveillance system as harboring 7–8 kDa PK-resistant fragments. As shown in Fig 6E, this thermolysin resistant PrP doublet of ~16 kDa is shared by brain material obtained from four other GSS alleles (Fig 6E)

### Origins and properties of the thermolysin-resistant PrP species

**Fig 7:**
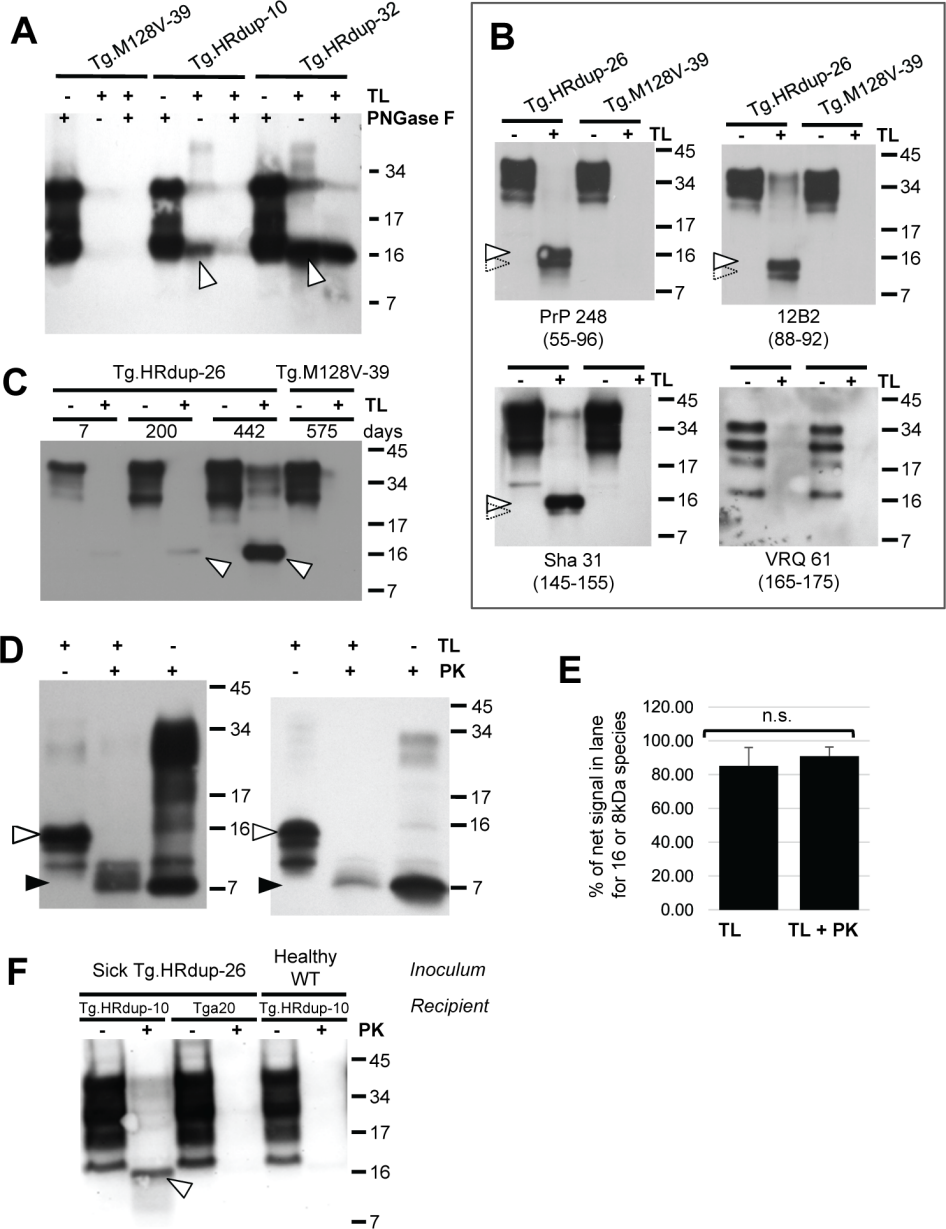
A 16 kDa thermolysin-resistant PrP signature in mouse brain material. A) 16 kDa thermolysin-resistant PrP signature (open arrow) found in Tg.HRdup-10 and -32 mice is not altered in mobility by PNGaseF treatment. Animal ages are line 16, 445d; line 10, 630d and line 32, 176d. A healthy TgM129V-39 control (684d) is also shown. B) The immunoreactivity of the 16 kDa species to various α-PrP antibodies was determined. PrP248 (residues 55–95), 12B2 (residues 88–92) and Sha31 (residues 145–152) reacted whereas VRQ 61 (res 165–175) did not. A second lower Mr species, suggesting TL-digestion generates ragged termini, by is indicated by an open arrow with a dotted perimeter. These antibody data suggest the larger TL-resistant species maps to residues 23–155 (mouse numbering scheme for WT PrP), which corresponds in turn to a molecular weight of ~14.7 kDa. C) Time-course of appearance of thermolysin-resistant HRdup PrP (indicated by open arrow); this signal increases as the animals age. Some residual full-length PrP of ~34 kDa can be noted at the fourth time-point. D) Double-digests to define a precursor-product relationship between 16 kDa TL-resistant and 8 kDa PK-resistant PrP species. Single and double digests of a 398d and 340d Tg.HRdup-26 brains. In each blot, the first lane represents 50 μg protein digested with thermolysin, second lane represents 100 μg protein digested with thermolysin and then digested with PK, with the major product migrating at 8kDa (open and black arrows, respectively). The third lane represents the sample digested only with PK but overloaded (100 μg load) to emphasize the 8kDa product. Heterogeneity, indicative of ragged termini, is apparent for these gel loadings, as per panel B. E) Quantitation of signal in TL and TL plus PK digests. Data represent digests of brain material from five Tg.HRdup-26 mice (range 340–398 d.) Left hand column represents percent signal in TL digest in the vicinity of 16 kDa versus net integrated signal in gel lane between 34 and 4kDa. Right hand column represents percent signal in TL plus PK digest from 12 to 4 kDa species versus net integrated signal in gel lane between 34 and 4kDa. Percentage values between single and double digests are not significantly different (n.s.). F) Low expressing Tg.HRdup-10 and overexpressing *tga20* (~6x) animals received an intracranial inoculation with brain homogenate from a sick Tg.HRdup-26 animal. Tg.HRdup-10 animals inoculated with healthy brain homogenate were euthanized after 400 days. Tg.HRdup-10 animals inoculated with clinical Tg.HRdup-26 brain homogenate succumbed to disease at 276±38 (SD) days. Thermolysin exposure of brain homogenates from inoculated animals is presented. Only Tg.HRdup-10 animals inoculated with Tg.HRdup-26 brain homogenate display the ~16 kDa thermolysin-resistant species (open arrow).

As anticipated, a TL-resistant 16 kDa species was also present in the brains of TgHR.dup-10 and Tg.HRdup- 32 mice in clinical phase of disease, but not in the control Tg.M128V-39 line (Fig 7A); electrophoretic mobility of the 16 kDa species was unaffected by the use of PNGase F, suggesting that the C-terminus must lie N-terminal to the glycosylation sites (Asn180 and Asn196); this assumption was validated by four antibodies with epitopes N-terminal to these two positions (Fig 7B). Our data exclude that the 16 kDa species corresponds to an un-glycosylated, thermolysin-resistant form of the C1 fragment produced by physiological endoproteolysis of PrP^C^—this is because two antibody epitopes (248 and 12B2) lie N-terminal to mouse PrP C1 N-termini at residues 109, 100 yet can detect the 16 kDa species and also because VRQ61 antibody that detects a C-terminal epitope present within C1 PrP nonetheless fails to detect the 16 kDa species. As the mouse 16 kDa species appears as a doublet with the PrP248 and 12B2 antibodies but less clearly so with Sha31, there may be raggedness at the C-terminus in the vicinity of the Sha 31 epitope, an effect which can be clearly observed using patient derived material (Figs 6E and 7B). Heterogeneous cleavage of PrP has also been noted *in vivo* during analysis of A117V, F198S and Q217R cases and following PK digestion of GSS cases harbouring the P102L mutation [52–54]. Levels of this species increased with age (Fig 7C). Sick Tg.HRdup mice could contain some immunoreactivity after thermolysin treatment indicative of full-length glycosylated PrP, but this was not the major species. Most notably, the 16 kDa species was detected at ages as young as 7 days in the brains of Tg.HRdup-26 mice (S12 Fig), underscoring occurrence preceding the 8 kDa fragment and hence a potential role as a precursor. With regards to accumulation, level of this species changed by 5-fold and net thermolysin-resistant PrP signal increased by 13.7-fold between pre-symptomatic (200d) and end-stage (442d) line 26 animals (Fig 7C).

We next used an in vitro double-digest experiment (Fig 7D) to explore the ability of 16 kDa thermolysin resistant PrP species to engender an 8 kDa PK resistant fragment. TL digests of 50 μg of protein from two aged Tg.HRdup-26 animals revealed a 16kDa fragment as the major species, along with two sub-molar species, again consistent with the inference of ragged fragment termini. As anticipated, an additional PK treatment after a TL digest yielded an 8 kDa species as the predominant product; in both cases this was accompanied by two sub-molar species, albeit with slower electrophoretic mobility than the predominant species (Fig 7D). We extended these analyses to three other aged Tg.HRdup-26 animals and quantified the percent signal in the vicinity of the 16 or 8 kDa species versus the complete integrated signal in the Mr range of 34–4 kDa. 16 kDa or 8 kDa species comprised > 80% signal in the TL or TL plus PK digests, respectively, and these percentages were not significantly different (Fig 7E). Thus, in an *in vitro* situation, the major product of a TL digest is efficiently converted after an additional PK digestion to a major product that has similar mobility to the major product of a PK digest; in short, the novel 16 kDa TL-resistant species can engender the 8 kDa PK-resistant hallmark species.

Concerning templated seeding and 16 kDa species, the inoculum from Tg.HRdup-26 mice that produced early clinical disease in Tg.HRdup-10 increased the level of 16 kDa species above genotypic controls treated with inoculum from healthy WT mice. No such species was detected in *tga20* mice (i.e. expressing WT mouse PrP) seeded with Tg.HRdup-26 inoculum (Fig 7F), suggesting an allelic barrier to seeding [37, 50, 51].

## Discussion

### Genetic versus transmissible aspects of GSS disease

Prion diseases can be sporadic, infectious or genetic; GSS, in particular, while caused by germline mutations, can also be infectious in certain lab settings. Mice expressing either WT murine PrP with a stop codon before the GPI anchor signal sequence or a natural I109 allele of WT bank vole PrP are reported to develop a spontaneous GSS-like syndrome [21, 55] but, for the most part, the goal of attaining GSS-like neuropathology and plaque deposition has been met by Tg mice that introduce mutations within the framework of mouse *Prnp* [56–58]. Conversely, P101L knock-in mice with 1x endogenous expression levels remain asymptomatic [59, 60], as do mice expressing human PrP with a P102L or A117V mutation [14, 61] and neither line are described as having spontaneous accumulation of pathognomonic 7–8 kDa PK-resistant fragment. This work presents a third transgenic model of GSS with spontaneous disease appearance where the human GSS mutation is used in the context of WT mouse PrP (the *Prnp*^a^ allele) but without synthetic epitope tags [62], thus joining Tg.P101L and Tg.A116V models [56, 57]. While PK-resistant PrP has not been observed in the brains of Tg.P101L mice or P101L knock-in mice [15, 32, 56, 59, 63], here we were able to detect robust levels of a pathognomonic 8 kDa PK-resistant fragment in two Tg lines with spontaneous disease. A similar low molecular weight PK-resistant PrP fragment present in A117V patients has been detected in the brains of sick Tg.A116V mice, though seemingly less prominent than observed in the HRdup expressing animals analyzed here [57] (Fig 2B–2E). An 8 kDa fragment is seen in transmissions from GSS tissue into bank voles [16], and in spontaneously sick Tg mice expressing bank vole PrP or GPI-anchorless mouse PrP [21, 55]. Association between the 8 kDa fragment and transmissibility is also present in our studies based on the HRdup GSS allele, where disease presentation in the lowest-expressor line, Tg.HRdup-10, was accelerated and levels of the 8 kDa PK-resistant fragment were enhanced over age-matched (asymptomatic) mice of the same transgenotype (Fig 2E, Table 1). This finding on species-barrier effects aligns with studies using other GSS models and a β-sheet enriched recombinant peptide approximating to the 8 kDa fragment [15, 32, 63, 64], but noting that transmissions into mice expressing P102L human *PRNP* can reveal different host-range properties than for mouse *Prnp* [65].

### Misfolding of HRdup and a 16 kDa thermolysin-resistant signature

What might be the *cis* effects of the 8 amino acid insertion upon folding of HRdup PrP? We failed to see significant distinctions between alleles in urea denaturation studies by monitoring five residues in the C-terminal globular domain of WT, M128V and HRdup PrP (Fig 4A and 4B, S7 Fig), suggesting the HRdup insertion does not affect the allele's PrP^C^-like global fold. Sequence inspection of HRdup reveals that the first β-sheet is split by the insertion, creating the potential for a second β strand separated by a short linker region (Fig 4C). As conversion from PrP^C^ to PrP^Sc^ involves an increase in β-content, one might speculate that HRdup has intrinsically more β-structure than a WT counterpart that could drive its conversion to a PK resistant form [5, 66]; while no additional β-sheets were noted in the ensemble measurements of recombinant protein preparations by NMR (Fig 4D and 4E), transient occupation of additional β-structure versus WT controls was noted in MD modeling following the trajectory of individual molecules (S9 Fig, S4 Table). In the HR of WT PrP there are four conserved glycines within three GxxxG motifs, while HRdup contains 6 equally spaced glycines within 5 of these motifs. GxxxG motifs were first identified as mediators of transmembrane helix-helix association [67]; there are indications from prion infections that these extracellular sequences may feature in uptake of prions in the replicative cycle [68]. *In vitro* work has shown that interruption of these GxxxG repeats decreases the PK resistance of recombinant PrP folded into a β-rich form. This finding has some parallels in allelic alterations in tandem repeats in Shadoo, a PrP family member with a HR lacking an N-terminal palindrome but instead composed solely of GxxxG repeats [69–71]. Further appraisal of the properties of GxxxG sequence motifs in PrP^C^’s HR may thus be fruitful. Beyond this, as the HR is only one part of PrP^C^'s natively disordered region and since perturbations were noted in sequences N-terminal to the insertion in MD simulations of monomers and in a dimer interface in *in silico* docking studies (S11 Fig), the question arises as to whether a larger region is impacted by the HRdup mutation. As a chemical probe, we employed thermolysin, an enzyme used previously to assess A116V PrP expressed in cell culture [72] and where the authors reported a greater resistance of this GSS associated allele to thermolysin relative to PK. However, to the best of our knowledge, the discrete N-terminal 16 kDa thermolysin-resistant species present at robust levels (and using the enzyme at 70°C [73]) is a previously unreported feature, being in line with the distinct molecular nature of GSS in that it differs from digestions of CJD and scrapie brain material.

**Fig 8:**
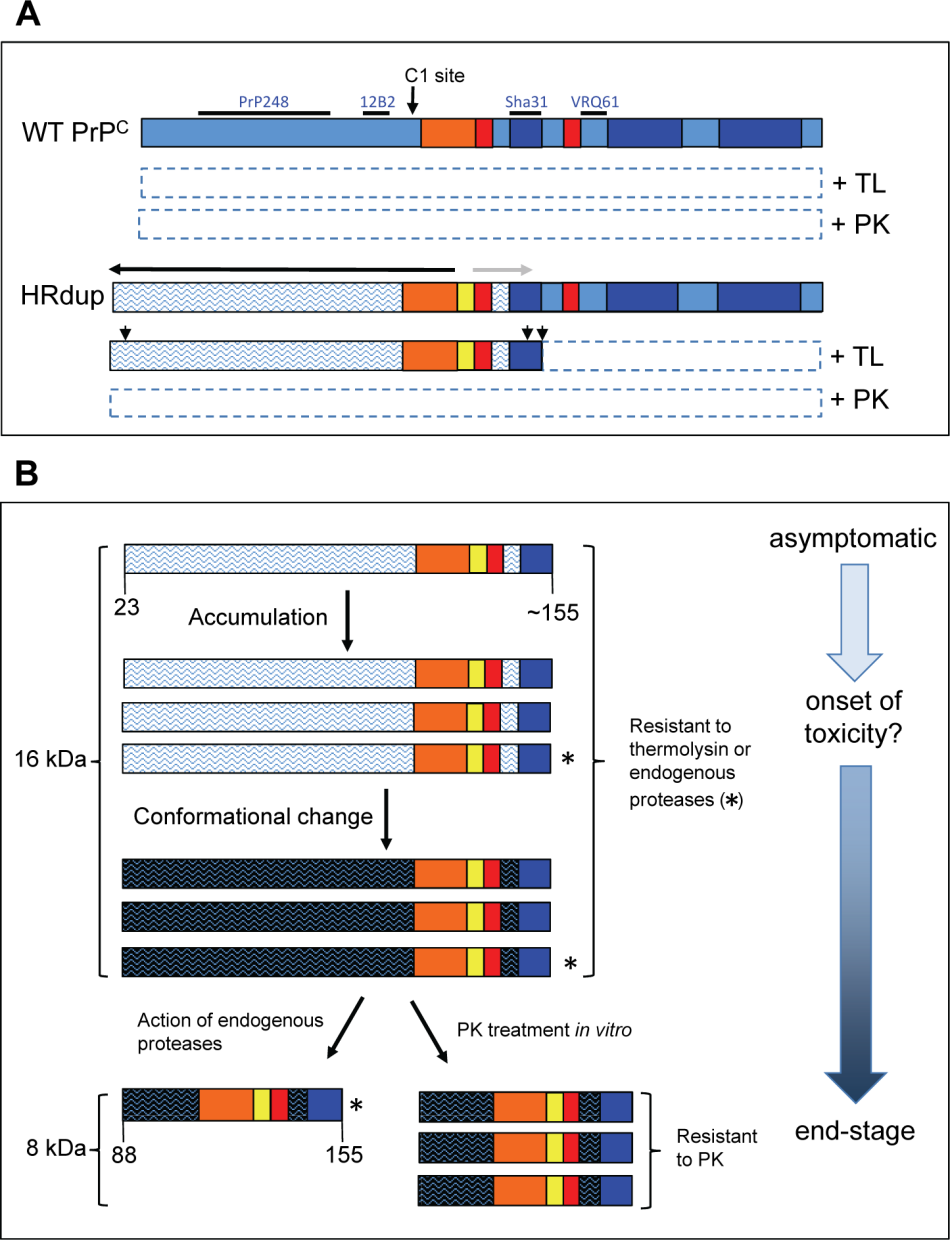
Ontogeny and properties of abnormal PrP species in Tg.HRdup mice. A) In vitro proteolysis products of HRDup and WT PrP derived in young animals. The hydrophobic region (HR) is presented in orange, helices in dark blue, beta strands in red and all other residues indicated by mid-blue shading. The approximate location of the epitopes of the anti-PrP antibodies used to map the boundaries of the protease resistant domains are indicated. In the presence of thermolysin (TL) and proteinase K (PK) WT PrP^C^ is completely degraded (indicated by dotted outline). In contrast, the disposition of HRdup PrP is different, with conformational effects of the 8-residue insert (yellow) insert spreading both in an N-terminal direction (horizontal black arrow) and in a C-terminal direction (horizontal grey arrow); these changes in conformation are indicated by the mid-blue shading of PrP^C^ being replaced by wavy lines. While being completely degraded by PK (like PrP^C^) HRdup can adopt a conformation whereby the N-terminal region is TL-resistant and hence only the C-terminal portion of the molecule is degraded by this protease. From gel analyses the cleavage sites may be heterogeneous ("ragged termini"); these termini have not been mapped in detail but are shown in a provisional manner by small vertical arrows. The action of C1 protease (large vertical arrow) immediately N-terminal to the HR would preclude the formation of 16 or 8 kDa protease-resistant species. B) Proposed evolution of protease resistant PrP species from HRdup PrP. For simplicity, ragged termini have been omitted from this schematic. The aging process is shown on the vertical axis. The signature 16 kDa thermolysin species of the HRdup PrP is already present in young mice, exhibits a slow accumulation with aging but remains PK sensitive. A similar species may accumulate spontaneously from endogenous protease action without the need for in vitro thermolysin digestion (asterisk). At a later stage in the disease course the 16kDa species are hypothesized to undergo a different conformational change (indicated by dark blue wavy fill) such that its C-terminal region (i.e. the central region of PrP) acquires resistance to endogenous proteases and accumulates; it also has the property of being resistant to PK digestion performed in vitro and yields the GSS signature 8 kDa PK-resistant fragment. The 8 kDa species can be amplified by templated refolding as shown in transmission experiments. Some full-length protease-resistant PrP may also be present at disease end-stage (e.g., Fig 7C, lane 6) but this is neither the predominant species nor consistently present and hence is not represented here. The presence of 16 kDa TL-resistant species in a number of GSS cases containing 7–8 kDa PK-resistant species (Fig 6E) suggest that this scheme may be generally applicable.

Multimerization and acquisition of protease-resistance often go hand-in-hand for prion diseases and here we note the HRdup PrP holoprotein has different assembly properties than M128V PrP (Fig 3) and that a self-aggregation determinant [74] lies within the boundaries of the 16 kDa species defined by epitope mapping. Moreover, unlike the natural C1 endoproteolytic fragment of PrP, the boundaries of 16 kDa thermolysin-resistant species do not preclude a precursor relationship to the 8 kDa form (Fig 8). Indeed, there is an interrelationship in the levels of 16 and 8 kDa species in i) different brain areas from the index case (Fig 6B), ii) in time-course analyses of normalized brain protein extracts of aging mice where the 16 kDa species occurs earlier than the 8 kDa fragment (Figs 2B, 7C) and iii) in sequential digest experiments (Fig 7D). Our data support a series of events wherein a fraction of full-length HRdup undergoes misfolding in the N-terminal domain such that it starts to assemble to multimers, becomes precipitable with PTA and yields a 16 kDa signature upon thermolysin digestion (Figs 2, 3, 6, 7 and 8). Enhanced accumulation of the 16 kDa species in inoculated Tg.HRdup-10 mice (Fig 7F) is also compatible with some templating and self-assembly capacity that may warrant further investigation. Over the course of time this conformationally altered form of PrP may yield 8 kDa PrP through the action of endogenous degradative processes (Figs 3A and 8) [38]. Conversely, the use of distinct proteases and antibody mapping, as well as its earlier appearance, exclude an alternative explanation that the 16 kDa PrP species detected by immunoblot reflect SDS-resistant dimers of 8 kDa PrP species.

### 16 kDa thermolysin resistant PrP and disease pathogenesis

The presence of the 16 kDa signature in four other GSS alleles and its increased levels in more pathologically affected areas of the brain from the index case (Fig 6A, 6B and 6E) both argue for intimate involvement in the disease process. In addition, the C-terminal boundary of the thermolysin-resistant species adjacent to the end of helix 1 offers an unexpected parallel to pathogenic stop codon mutations such as PrP145X, where structural biology and transmission properties have been studied in depth [75–77]. Finally, Tg.HRdup-32 mice succumb to spontaneous disease earliest of all the Tg lines described here and accumulate 16 kDa thermolysin resistant PrP but not the 8 kDa PK resistant fragment (and, hence, cannot accelerate disease in recipient animals), so a straightforward inference from these net data is that the misfolded form of PrP revealed by thermolysin digestion has neurotoxic activity. Hypothetical toxic forms of PrP (PrP^L^) have been inferred for prion infections, with clinical disease emerging when levels of PrP^L^ transcend a threshold; in this scheme, autocatalytic propagation of PrP^Sc^ ("Phase 1") precedes toxicity ("Phase 2") [78]. We suggest that a permutation of these concepts may come close to representing the GSS pathogenic process; specifically, the potentially toxic species could start to accumulate early on and be diagnosed in tissue by thermolysin treatment as the 16 kDa species. The later disease process may be characterized by two events. First, noting that the level of the 16 kDa thermolysin-resistant species rises with chronological age in Tg mice, levels of toxic misfolded forms of PrP may transcend a threshold needed for clinical manifestation. Second, the 8 kDa PK-resistant species that is adept at autocatalytic propagation events—"infectivity", as measured in experimental transmissions from human source material in bank voles [16] or from Tg mouse material (Fig 2E)—begins to rise. Delayed occurrence of the 8 kDa species may reflect its derivation from the form of misfolded PrP represented by thermolysin resistance. This scheme of pathogenesis is summarized in Fig 8B. While full-length thermolysin-resistant forms of PrP were present at end-stage in Tg mice (Fig 7A–7C) and might also have toxic potential, levels of full-length thermolysin-resistant forms of PrP were unremarkable in human GSS tissue (Fig 6A, 6D and 6E) and hence were not considered an obligatory item in pathogenesis. Future studies to inventory the levels of abnormally folded full-length PrP by use of PTA (Fig 6B and 6C) or by detergent insolubility, may be useful to probe and refine this new view of pathogenesis.

In terms of accumulation of 16 kDa thermolysin-resistant PrP and toxicity thresholds, for the Tg.HRdup-26 line, the 16 kDa signature is apparent at almost the very time PrP transcript levels increase in rodents above the levels present in embryos, this transition correlating with neuronal precursor cells ceasing proliferation and beginning to differentiate [79–82]. Clinical disease is scored 400 days later in Tg.HRdup-26 mice and ~170 days later for Tg.HRdup-32 mice. These data beg the question of whether an early start to the accumulation of abnormal PrP species resulted in a corresponding early transit across a threshold for manifestation of clinical symptoms. Of interest, the index case had psychiatric events in his twenties and is reported to have a son affected with an autism spectrum disorder condition (Asperger's) [24]. Both WT PrP^C^ and HRdup PrP undergo physical interactions with DPP6 [83, 84], a type II membrane protein [85] that controls dendritic morphogenesis and one wherein gene disruptions link to different neurodevelopmental disorders [86–88]. Thus, a number of fascinating possibilities may emerge from further investigation of this kindred. More broadly, the Tg.HRdup mice comprise a new tool for structural biological investigations to investigate changes in PrP's N-terminal region [6] and links to neurotoxicity. Given that other GSS mutants share this chemical signature, there may an opportunity to understand common disease mechanisms in GSS and, pending the generation of new molecular probes for this form of PrP, perhaps more common CNS disorders as well.

## Materials and methods

### GSS patients and material classification, brain samples, and PRNP gene sequencing

DNA was extracted from frozen brain tissues in all cases, and genotypic analysis of the *PRNP* coding region was performed as described [89–91]. On the basis of diagnostic pathology, immunohistochemistry, and western blot examination of 3 brain regions (including frontal, occipital and cerebellum cortices) with mAb 3F4 and 1E4, the pathogenic PrP^Sc^ was classified as described previously [31, 92–95]. Coronal sections of human brain tissues were obtained at autopsy and stored at -80°C. Three 200–350 mg cuts of frontal (superior and more posterior middle gyri) cortex were taken from each brain and used for molecular analyses. The other symmetric cerebral hemisphere was fixed in formalin and used for neuropathological classification of prion disease using histological and immunohistochemical analysis of samples from 16 anatomical areas and NPDPSC’s standard protocols [93, 96, 97]. We based the classification on the molecular characteristics of PrP^Sc^ on western blots developed with a panel of antibodies as described previously [93, 98, 99] to exclude GSS cases with a 21 kDa PK-resistant fragment. This criterion, as well as DNA sequencing, allowed the classification of the included cases with pathognomonic 8 kDa fragments as follows: A117V-129V (age 35, disease duration 60 months), P102L-129M (age 37, disease duration 106 months), H187R-129V (age 42, disease duration 108 months) and F198S-129V (age 59, disease duration 120 months). For the index case, we used frozen postmortem tissue from different brain areas, as described in the Figure legends. Healthy brain tissue as well as MV1 and VV2 CJD samples were similarly classified. Cortical samples were used to make homogenates.

### Animal husbandry and inoculations

Transgenic mice were generated using a modified half-genomic construct and standard procedures [34, 100]. Animals were housed in groups of up to five under a 12 h light/dark cycle with food and water *ad libitum*. Tg.CRND8, Tg.TauP301L and *tga20* mice have been described previously [34, 101, 102]. Inoculations were performed by intracerebral injection of 30 μl of 1% (wt/vol) brain homogenate.

### Western blotting and limited proteolysis

Brain hemispheres were homogenized in cold PBS by passage through successively larger gauge needles. Whole brain extract was subjected to 10% Tricine-SDS-PAGE and transferred to PVDF membranes (Millipore) using Tris-gly transfer buffer with 20% methanol in the Mini Trans-Blot Electrophoretic Transfer Cell (BioRad) or the XCell Blot Module (Invitrogen). Primary antibodies used were: Sha31 (α-PrP; Spi-bio), 12B2 (α-PrP; from Dr. J. Langeveld) and PrP248 and VRQ61 (α-PrP; from Dr. H. Rezaei). Secondary antibodies used were horseradish peroxidase conjugated goat α-mouse (Bio-Rad). For enzymatic digestion, 250 μg (PK) or 50 μg (thermolysin) of protein was incubated for 1 h at 37°C (PK) or 70°C (thermolysin) with 10 μg/ml PK (Roche) or 50 μg/ml thermolysin (Sigma) in 250 μl. For sequential digests, samples were methanol precipitated after thermolysin treatment, re-suspended in PBS and then digested with PK. 5 mM PMSF (PK) or 10 mM EDTA pH 8 (thermolysin) was used to stop the reaction and samples were centrifuged at 20,800 x g for 1 h at 4°C. Pellets were resuspended in sample buffer containing 50 mM DTT. For removal of carbohydrates, 20 μg of protein was incubated overnight at 37°C with 100 U PNGase F (New England Biolabs) in a volume of 20 μl according to manufacturer's instructions. Molecular weight markers are SeeBlue Plus 2 pre-stained standards (Invitrogen).

### Histopathological analysis

**Mice:** Sagittal sections were fixed in 10% phosphate buffered formalin and embedded in paraffin. Hematoxylin and Eosin staining was done as previously described [103]. For immunodetection, slices were heated to 121°C in 10 mM citrate buffer and allowed to cool to room temperature. Staining for PrP^Sc^ was then accomplished by treatment with formic acid and 4 M guanidine thiocyanate before an overnight incubation with biotinylated SAF83 (Cayman Chemicals) or PrP248. GFAP immunodetection was accomplished by subsequent incubation in 3% peroxide and overnight incubation with a biotinylated primary antibody cocktail (BD Biosciences; 556330). These slices are counterstained with Mayer’s hematoxylin. ***Index case:*** Brain tissue was obtained at autopsy 21 hours after death. The tissue was partly frozen at -85°C and partly fixed in buffered formalin. The tissue was processed to blocks and embedded in paraffin 14 days after autopsy. Five micrometer sections were stained with hematoxylin and eosin, Periodic Acid-Schiff, and immunohistochemistry was performed for prion protein (12F10 1:3000 Cayman), amyloid beta (Anti B-Amyloid 17–24 (4G8) 1:20000 BioLegend), tau (AT8, 1:2500 Leica), and P62 (Anti-SQSTM1 1:200 Abcam).

### PTA precipitation

An equal volume of 4% Sarkosyl was added to 1 mg of a 20% brain homogenate (PBS) and homogenized by passage through a 25g needle. A stock solution of PTA was added to have a final concentration of 2% PTA and the sample was allowed to incubate at 37°C for 16 h with 1200 rpm shaking [39]. The sample was centrifuged at 16,000 x g for 30 minutes and the supernatant was removed. The pellet was resuspended in 50 μl 0.2% Sarkosyl and then brought to 1000 μl using 2% Sarkosyl. PTA was again added to a final concentration of 2% and the sample was incubated at 37°C for 1 h. Following a second 16,000 x g spin, the pellet was resuspended in 0.2% Sarkosyl for downstream analysis.

### Sucrose gradient ultracentrifugation

Linear 10–45% sucrose gradients were prepared by layering 375 μl of increasing concentrations (5% steps) of sucrose (in PBS, pH 7.4 and 1% Sarkosyl) in OptiSeal polypropylene tubes (Beckman Coulter). Gradients were linearized by incubation overnight at 4°C. 250 μg of brain homogenate was brought to 300 μl in PBS (pH 7.4) containing 2% Sarkosyl and layered on top. Samples were centrifuged at 268,000 x g for 73 minutes at 4°C using a swinging bucket rotor and eight fractions were collected from the bottom of the tube. Equivalent volumes of each fraction were then interrogated for the presence of PrP by western blot.

### NMR spectroscopy and analysis

The genes moPrP_118-231_, moPrP_118-231_ M128V, and moPrP_118-231_ HRdup, were synthesized by DNA2.0 with codon optimization. The N terminus of all three proteins had a 6xhis tag with a TEV cleavage site for removal. Protein sequences are listed below:

### Mouse PrP(118–231) M128V HRdup, 143aa

HHHHHHGASTGGQQGENLYFQGAVVGGLGGYVLGGLGGYVLGSAMSRPMIHFGNDWEDRYYRENMYRYPNQVYYRPVDQYSNQNNFVHRCVNITIKQHTVTTTTKGENFTETDVKMMERVVEQMCVTQYQKESQAYYDGRRSSG

### Mouse PrP(118–231) M128V, 135aa

HHHHHHGASTGGQQGENLYFQGAVVGGLGGYVLGSAMSRPMIHFGNDWEDRYYRENMYRYPNQVYYRPVDQYSNQNNFVHRCVNITIKQHTVTTTTKGENFTETDVKMMERVVEQMCVTQYQKESQAYYDGRRSSG

### Mouse PrP(118–231) WT, 135aa

HHHHHHGASTGGQQGENLYFQGAVVGGLGGYMLGSAMSRPMIHFGNDWEDRYYRENMYRYPNQVYYRPVDQYSNQNNFVHRCVNITIKQHTVTTTTKGENFTETDVKMMERVVEQMCVTQYQKESQAYYDGRRSSG

Vectors were transformed into BL21(DE3) cells. The proteins were expressed and purified based on the methodology previously described for the expression of human PrP [104].

For urea denaturing experiments, the samples were prepared the same as described previously [2]. Whilst for two-dimensional (2D) ^1^H-H^1^ NOESY experiment, 2 mg protein was dissolved in 500 μl solution that was made up with D_2_O (99.9%, Cambridge Isotope Laboratories), 10 mM sodium acetate, and 0.3 mM DSS-d_6_; pD was adjusted to 5.3 using 2 M DCl. The sample was flash frozen, lyophilized and re-suspended using 500 μl D_2_O (99.996%, Cambridge Isotope Laboratories) to minimize the H_2_O signal within the spectra thereby providing a clear view of β-sheet-correlating signals.

NMR experiments were performed on an 800 MHz Varian INOVA NMR spectrometer at 25°C. One-dimensional (1D) proton NMR spectra were acquired for urea denaturing experiments with 256 transients, with a spectral width of 15.0 ppm and a time delay of 2.5 s; while two-dimensional (2D) H^1^-H^1^ NOESY spectra were acquired with 64 transients, 768 increments, spectral widths of 12.0 ppm for both proton dimensions. The time delay was 1.5 s and the mixing time was 100 ms. All NMR spectra were processed with VnmrJ software v4.0 (Varian Inc.) and line broadening of 0.5 Hz was applied. Further data processing and analysis for both urea denaturing experiments and 2D experiments are the same as previously described [105].

### Modeling of the insert structure

The insert model HRdup was built using the SWISS-MODEL homology modelling server [106]. The moPrP 23–239 sequences with the M128V polymorphism were modified by insertion of a LGGLGGYV sequence between V128 and L129 and uploaded to SWISS-MODEL. The server template search and alignment with BLAST and HHBlits software [107, 108] were performed. Direct insertion of a LGGLGGYV between V128 and L129, or GLGGYVLG between G130 and S131 with target-template alignments were also attempted. Those constructs tended to exhibit similar conformations as in the model we adopted for analysis (S3 Table). Both the global and per-residue model quality were assessed using the QMEAN scoring function [109]. Two scores were evaluated: the global model quality estimation (GMOE; scores closest to one indicate the highest quality) and score composite scoring function to estimate the global and local model quality (QMEAN4; the highest negative scores indicate a higher local per-residue reliability of the model). The homology search for mouse PrP sequence with insert produced 285 templates, from which 10 were chosen to build models. These included PDB ID codes 4MA8, 4MA7, 2L39, and 2L1H (MoPrP) and 4KML, 1QLZ (HuPrP), see S3 Table. The best matching models chosen based on the range, sequence identity, and coverage, included constructs 2L39 with identity 98.25%, GMQE score 0.41 and QMEAN4 score -4.03; 2L1H with identity 99.11%, GMQE score 0.45, and QMEAN4 score -1.94; and 4MA7 with identity 99.12%, GMQE score 0.46 and QMEAN4 score -1.56. The coordinates of the eight amino acid insert model based on the 2L39.pdb construct were uploaded and prepared for the simulations. The best-matching 3D model of HRdup constructed by the SWISS-MODEL tool initially represented a L124-D226 sequence. The N-terminal fragment G89-G123 was added to this initial model using the Accelrys VS software (*Dassault Systèmes BIOVIA*, *Discovery Studio Modeling Environment*, *Release 2017*, *San Diego*: *Dassault Systèmes*, *2016*). The choice of pdb structure and the length of the sequence for the control M128V structure without the insert were based upon the best-matching model 2L39.pdb. C-terminal fragment 227–232 was removed to match the HRdup model, and fragment 89–117 and polymorphic mutation M128V were introduced using the Accelrys VS. In all simulation runs, the C-terminal and N-terminal extremities of the main chains were kept charged (−COO- and −NH3+), whereas all other titratable amino acids were assigned their canonical state at pH 4.5 with the PropKa server software [110].

### Molecular dynamics simulations

The HRdup and M128V constructs were subjected to minimizations, equilibrations and production molecular dynamics (MD) simulations in Gromacs v 4.5.3 package with OPLS forcefields [111, 112]. Starting models were minimized *in vacuo* for 10000 steps of steepest descent minimization. Then the models were solvated in single point charge extended (SPC/E) rectangular periodic water box, after which Cl^−^ or Na^+^ ions were added to neutralize the systems. Subsequent solvent minimizations with decreasing position restraints (K_posre_ = 1×10^5^, 1×10^4^, 1000, 100, 10 and 0 kJ mol^−1^ nm^−2^) on non-hydrogen protein atoms have been made to relax solvent and protein. Subsequent heating with the Berendsen thermostats from 0 K to 310 K and NPT equilibration with adjustment of solvent density to 1 g/cm^3^ followed the minimizations. The last equilibration step and the production simulations were conducted at 310 K temperature and at a pressure of 1 atm with isotropic pressure coupling (NPT ensemble). The bond lengths were restrained with the LINCS algorithm with a fourth order of expansion. The short-range electrostatic and van der Waals interactions cut-off radii were equal to 14 Å each. Long-range electrostatic interactions were treated with the particle-mesh Ewald (PME) summation with grid spacing of 0.135 nm for the fast Fourier transform and cubic interpolation. The simulations were performed for 20 ns for each system; 1 fs time steps were employed, and snapshots saved every 20 fs in order to analyze the essential collective dynamics. For each of the HRdup and M128V constructs, the MD simulations were duplicated from the same starting coordinates, using different starting velocities of atoms. The corresponding MD trajectories are denoted as “I” and “II” in the discussion.

### Analysis tools

To analyze the PrP constructs from MD trajectories, their secondary structure content, numbers of hydrogen bonds and salt bridges, contact maps, and solvent accessible areas (SASA) have been calculated using scripts implemented in Gromacs [112, 113] and VMD [114] packages. Final SASA analysis was made according to solvent exposure level defined in [115]. Snapshots from trajectories and graphical representation of models was done with VMD or Accelrys VS [115]. Protein docking and predictions of residues involved in oligomer contacts for representative snapshots from M128V and HRdup trajectories were made through the InterEVDock server integrated in the RPBS Mobyle portal [116].

### Essential collective dynamics

To analyze and compare dynamics of PrP alleles in greater depth we employed the novel essential collective dynamics (ECD) method [45, 47, 117–120]. The method stems from the statistical-mechanical analysis of the generalized Langevin dynamics of proteins [117, 119], according to which persistent correlations between atoms’ motion in the protein can be determined from principal eigenvectors of the covariance matrix of a protein’s MD trajectory. A suite of dynamics descriptors has been derived within this framework, including in particular the main-chain flexibility profiles and pair correlation maps [118, 120]. Previously the method has undergone an extensive validation against NMR-derived (117, 119) and X-ray based structural data [47, 118, 120], and was demonstrated to predict accurately the main-chain flexibility, pair correlations, and other dynamics trends from short fragments of MD trajectories. In this work, the ECD main chain flexibilities and pair correlation maps are obtained for the HRdup and M128V constructs using the techniques described in detail elsewhere [45, 47, 120].

### Protein docking

Representative conformations from the four production MD trajectories were used as templates for protein docking on the InterEVDock server integrated in the RPBS Mobyle portal [121]. 10,000 decoys were created, scored and clustered resulting in 10 models for each of three scoring methods (InterEVScore, SOAP_PP atom-based statistical potential, and FRODOCK). Conservation of residues was assessed with the rate4site.

### Ethics statement

Procedures for the index case have already been described [24]. All other procedures were performed under protocols approved by the Institutional Review Board at Case Western Reserve University. In all cases, written informed consent for research was obtained from the patient or next of kin and the material used had appropriate ethical approval for use in this project. All patient data and samples were coded and handled according to NIH guidelines to protect patient identities. For animal studies, all protocols were in accordance with the Canadian Council on Animal Care and were approved by the Animal Care and Use Committee at the University of Alberta (AUP00000356).

## Supporting information

S1 Fig The cellular location of HRdup is comparable to that of M128V PrP. A) RK13 cells were transiently transfected with pcDNA3 vectors encoding WT, M128V and HRdup PrP. Lysates without PNGase F treatment run in Tris-glycine gels showed a similar degree of glycosylation. Lysates after PNGase F treatment showed similar amounts of full-length (FL) PrP, as well as similar amounts of C1 endoproteolytic cleavage products. B) RK13 cells were transiently transfected with WT, M128V and HRdup PrP using a pcDNA3 vector. The biotinylation reagent is impermeable to the cell membrane. Total PrP (T) and biotinylated PrP (S, cell-surface) are loaded side-by-side and electrophoresed using a Tris-glycine buffer. HRdup is comparably accessible to the biotinylation reagent as control PrP. C) Transfected cells as per panel A were analyzed for cell-surface PrP without permeabilization (top row) or after permeabilized with Triton X-100 ("+TX"; bottom row) to analyze intracellular PrP. No qualitative difference in the localization of the three proteins was observed (n = 6). All images represent a merge of Hoechst (nuclei, blue) and Alexa fluor 594 (red) signals. Conjugated secondary antibody was used with SAF83 primary antibody (both 1:2000). 40x magnification.
(TIF)

S2 Fig Histopathological examination of Tg.HRdup-10 and Tg.HRdup-32 animals. *Upper set of panels*. Photomicrographs of sagittal sections of a sick Tg.HRdup-10 mouse (585d). Immunostaining for PrP was performed after treatment of the slices with formic acid, slices destined for examination with H&E or GFAP were left untreated. The hippocampus (A-C), thalamus (D-F), cortex (G-I) and cerebellum (J-L) are shown. PrP^res^ immunostaining is absent the hippocampus, thalamus and cortex but is present in the cerebellum. Vacuolation is found in the hippocampus, thalamus and cerebellum. GFAP immunostaining is restricted to the cerebellum. Scale bar = 100 μm. Lower set of panels, photomicrographs of sagittal sections of a sick Tg.HRdup-32 mouse (162d) showing spongiform change. M, P cortex, N and Q cerebellum and O and R, hippocampus. H & E (M-O) and GFAP immunostains (P-R) are shown. Scale bar = 100 μm for cortex and cerebellum, 500μM for hippocampus. Insets (squares, O, R) show expanded views of spongy change but mild astrocytic activation.
(TIF)

S3 Fig Histopathological examination of Tg.M128V-39 animals. Photomicrographs of sagittal sections of healthy, aged Tg.M128V-39 mouse (576d). Immunostaining for PrP was performed after treatment of the slices with formic acid, slices destined for examination with H&E or GFAP were left untreated. The hippocampus (A-C), thalamus (D-F), cortex (G-I) and cerebellum (J-L) are shown. Vacuolation, PrP^res^ and GFAP immunostaining are absent in all regions in the brains of these mice. Scale bar = 100 μm.
(TIF)

S4 Fig Lack of myopathy and neuropathy in Tg.HRdup-26 mice. H&E stain of cross-section of quadriceps from aged WT (211d, A) and a Tg.HRdup-26 mouse (437 d, B). Scale bars 50 μm. Toluidine blue staining of a cross section of sciatic nerve from an aged *Prnp*^*0/0*^ (446d, C) showing irregular fibre diameters. A sick Tg.HRdup-26 mouse (437d, D). Scale bar = 25 μm.
(TIF)

S5 Fig Brain homogenate from sick Tg.HRdup animals induces early pathological changes in mice expressing homologous PrP. Low expressing Tg.HRdup-10 animals and *tga20* (overexpressing WT PrP ~6-7x) animals received an intracranial inoculation with brain homogenate from a sick Tg.HRdup-26 animal or a healthy WT animal. The onset of clinical disease and pathological change was accelerated in the animals receiving the HRdup brain homogenate. A) Kaplan-Meier death curve for animals in the study. n = 4 for all groups. *tga20* animals, with the exception of one that had to be euthanized due to intercurrent illness, were euthanized after 460 days. Tg.HRdup-10 animals inoculated with healthy brain homogenate were euthanized after 400 days. Tg.HRdup-10 animals inoculated with clinical Tg.HRdup-26 brain homogenate succumbed to disease at 276±38 days. B) Immunohistochemistry of the cerebellum of a Tg.HRdup-10 (top row) and *tga20* animal (middle row) inoculated with the brain homogenate from a sick Tg.HRdup-26 animal or Tg.HRdup-10 animal inoculated material from a healthy WT animal (bottom row). i, iv, vii, PrP^Sc^ immunoreactivity with SAF83; ii, v, viii, H&E stain; iii, vi, ix: GFAP immunoreactivity. Scale bar = 75 μm. Note that only animals that succumbed to illness (and harboring protease resistant PrP) display PrP immunoreactivity, vacuolation and astrocytic gliosis associated with prion disease.
(TIF)

S6 Fig 1D NMR spectra for three PrP alleles. 1D ^1^H NMR full spectra are presented for HRdup, M128V and WT PrP (top to bottom).
(TIF)

S7 Fig 1D NMR spectra of urea titrations for three PrP alleles. 1D ^1^H NMR experiment of WT (A), M128V (B) and HRdup PrP (C) titrated with urea from 0 to 10M. Upper panels show stacked plots of the aromatic region of spectra, ranging from 5.2 to 6.7ppm (resonances of Y162H*ε*, Y161Hα, F197Hα and Y217Hδ are indicated) while lower panels show stacked plots of the methyl region of spectra, ranging from -0.14 to 0.86 ppm (resonance of I181Hγ2 is indicated).
(TIF)

S8 Fig Complete 2D NMR spectrum. Full spectra of 2D ^1^H-^1^H NOESY of HRdup (A) and M128V PrP (B) are presented.
(TIF)

S9 Fig Secondary structure evolution in MD simulations. Shown are data from the M128V-I system (A), M128V-II system (B), HRdup-I system (C), and HRdup-II system (D). The total simulation time was 20 ns. Along the vertical axes, N-terminals are on the top and C-terminals are on the bottom. The legends underneath indicate the structure-color associations: T–turn (cyan), E–extended β conformation (yellow), B–isolated β bridge (dark yellow), H–α-helix (purple), G– 3-10-helix (blue), I–π-helix (red), and C–coil (white). The figures were obtained with the VMD package (43).
(TIF)

S10 Fig ECD correlation maps. These are calculated for side-chain atoms (above the diagonal) and main-chain atoms (below the diagonal) in M128V-I system (A), HRdup-I system (B), M128V-II system (C), and HRdup-II system (D). High levels of correlations are indicated with red and yellow, medium levels are indicated with green and light-blue; and low levels with dark blue. Some of the dark blue lines in the side chain correlation maps correspond to glycine (no side chains) and alanine (short side chains) residues, which were excluded from the side-chain analysis.
(TIF)

S11 Fig Docking analysis. Close-up of predicted homodimers M128V-I (A) and HRdup-I (B), and heterodimer M128V-I HRdup-I (C). The residues on the binding interface are shown with lines and translucent surfaces colored according to the electrostatic charge (translucent pink–negative charge; translucent blue–positive charge; white–neutral). In the display of the first chain in the dimers, α-helices are indicated with opaque red color; β-strands with light blue; random coils with grey; and turns or helix disruptions with green.
(TIF)

S12 Fig A 16 kDa thermolysin-resistant PrP species is present in neonatal Tg.HRdup-26 mice. Upper panel, western blot analysis of brains of eight neonatal animals (A-H) from the Tg.HRdup-26 line. Antibody is Sha31. The four left-hand samples have the thermolysin-resistant species. Lower panel, corresponding PCR analyses of genomic DNA from eight neonatal mice with reaction products displayed on SYBR safe stained agarose gel. The diagnostic fragment for the PrP transgene migrates at 550 bp.
(TIF)

S1 Text Supplementary Methods and tables. (DOCX)

S1 Table Pathology in mouse Tg.HRdup lines. (DOCX)

S2 Table *m* values Table for 5 PrP residues in a urea denaturation experiment. (DOCX)

S3 Table The results of SWISS-MODEL template library search with BLAST and HHBlits. (DOCX)

S4 Table Time-averaged β-content occupancies in four MD trajectories. (DOCX)

S5 Table Per residue solvent accessible surface area (SASA). (DOCX)

S6 Table Total SASA for two trajectories of M128V and HRdup PrP alleles. (DOCX)

S7 Table Residue pairs with robust inter-correlations for Cα atoms indicate PrP regions of the strongest dynamic correlations. (DOCX)

S8 Table Residues in M128V and HRdup monomers predicted to be involved in dimer contacts. (DOCX)
