# Supplementary figures and images for: A novel Gerstmann-Sträussler-Scheinker disease mutation defines a precursor for amyloidogenic 8 kDa PrP fragments and reveals N-terminal structural changes shared by other GSS alleles
Source: PLoS Pathog. 2018 Jan 16;14(1):e1006826. doi: 10.1371/journal.ppat.1006826 (PMC5786331; doi:10.1371/journal.ppat.1006826)

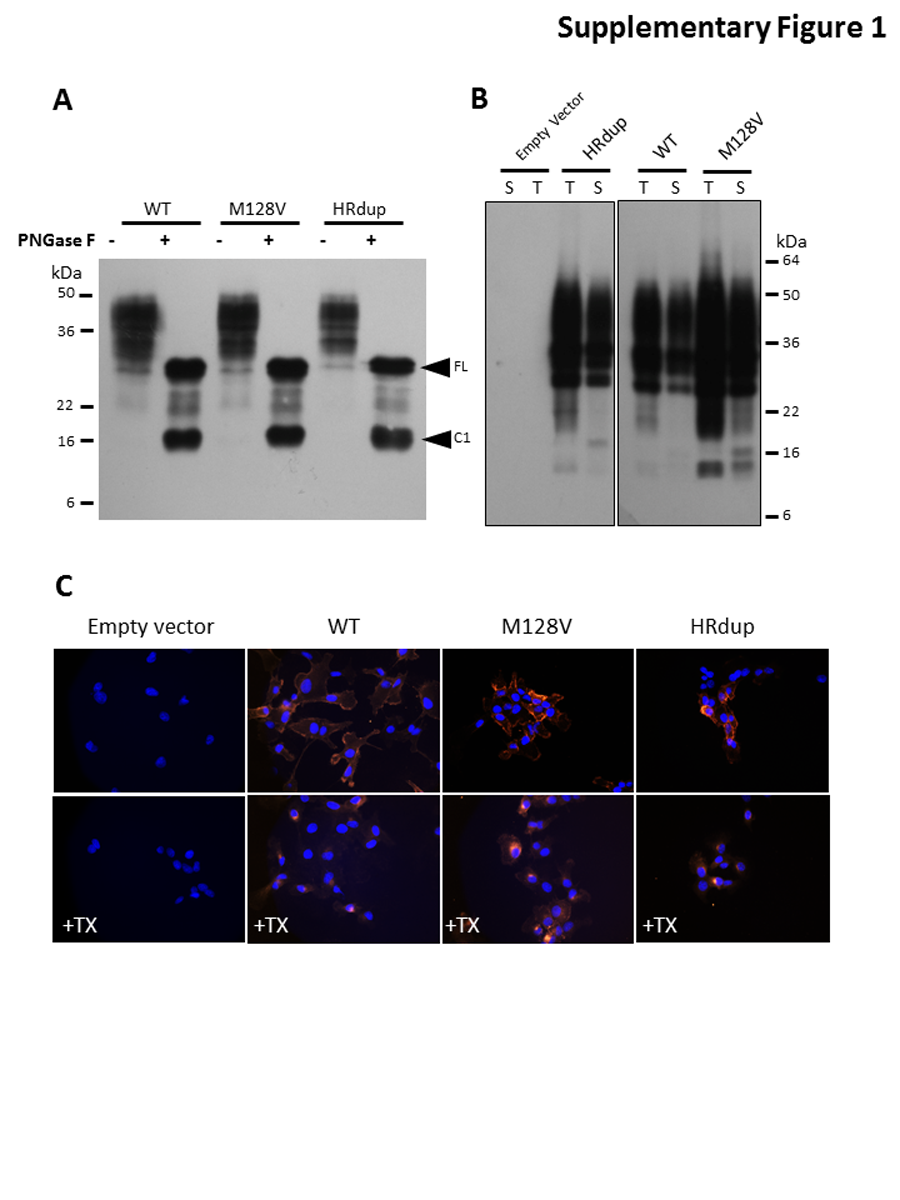

Supplement: S1 Fig — A) RK13 cells were transiently transfected with pcDNA3 vectors encoding WT, M128V and HRdup PrP. Lysates without PNGase F treatment run in Tris-glycine gels showed a similar degree of glycosylation. Lysates after PNGase F treatment showed similar amounts of full-length (FL) PrP, as well as similar amounts of C1 endoproteolytic cleavage products. B) RK13 cells were transiently transfected with WT, M128V and HRdup PrP using a pcDNA3 vector. The biotinylation reagent is impermeable to the cell membrane. Total PrP (T) and biotinylated PrP (S, cell-surface) are loaded side-by-side and electrophoresed using a Tris-glycine buffer. HRdup is comparably accessible to the biotinylation reagent as control PrP. C) Transfected cells as per panel A were analyzed for cell-surface PrP without permeabilization (top row) or after permeabilized with Triton X-100 ("+TX"; bottom row) to analyze intracellular PrP. No qualitative difference in the localization of the three proteins was observed (n = 6). All images represent a merge of Hoechst (nuclei, blue) and Alexa fluor 594 (red) signals. Conjugated secondary antibody was used with SAF83 primary antibody (both 1:2000). 40x magnification. (TIF) [file ppat.1006826.s001.TIF]

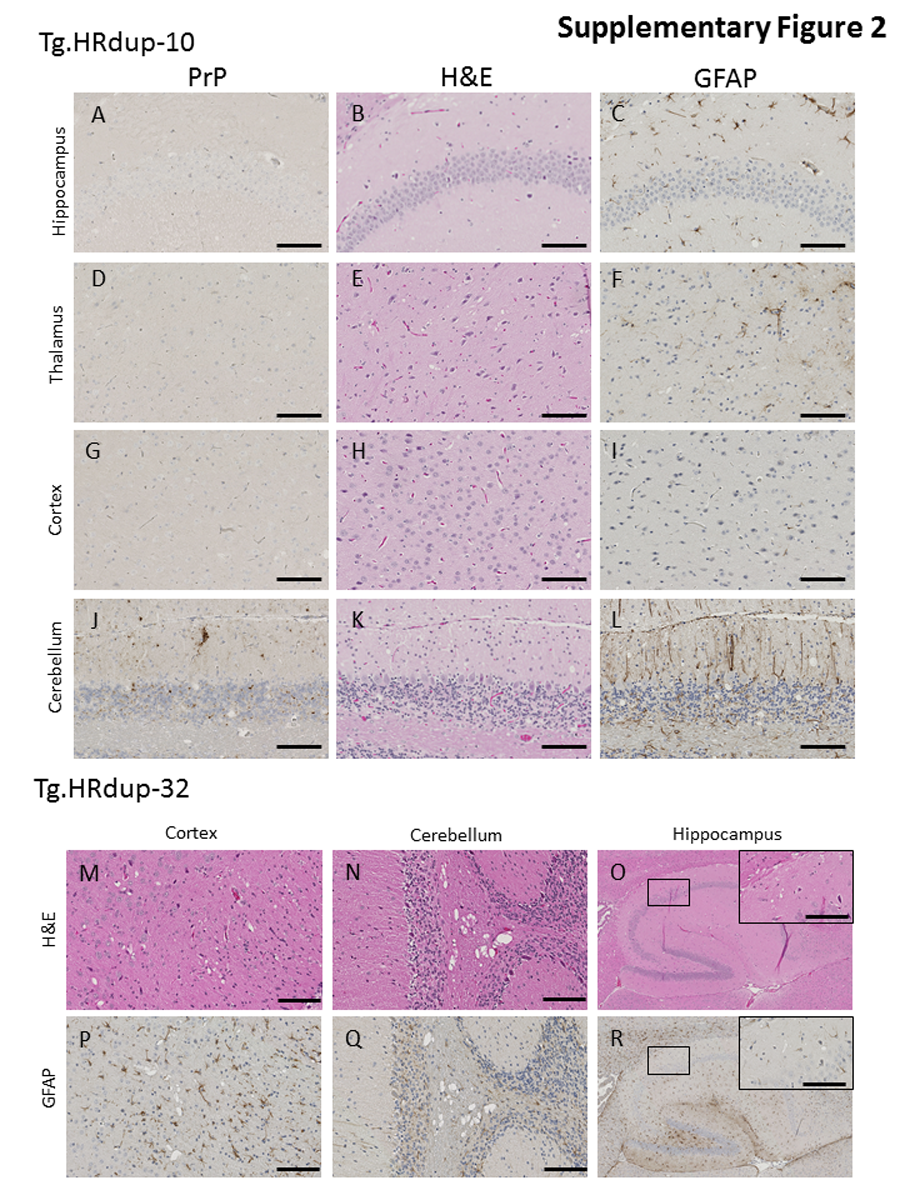

Supplement: S2 Fig — Upper set of panels. Photomicrographs of sagittal sections of a sick Tg.HRdup-10 mouse (585d). Immunostaining for PrP was performed after treatment of the slices with formic acid, slices destined for examination with H&E or GFAP were left untreated. The hippocampus (A-C), thalamus (D-F), cortex (G-I) and cerebellum (J-L) are shown. PrPres immunostaining is absent the hippocampus, thalamus and cortex but is present in the cerebellum. Vacuolation is found in the hippocampus, thalamus and cerebellum. GFAP immunostaining is restricted to the cerebellum. Scale bar = 100 μm. Lower set of panels, photomicrographs of sagittal sections of a sick Tg.HRdup-32 mouse (162d) showing spongiform change. M, P cortex, N and Q cerebellum and O and R, hippocampus. H & E (M-O) and GFAP immunostains (P-R) are shown. Scale bar = 100 μm for cortex and cerebellum, 500μM for hippocampus. Insets (squares, O, R) show expanded views of spongy change but mild astrocytic activation. (TIF) [file ppat.1006826.s002.TIF]

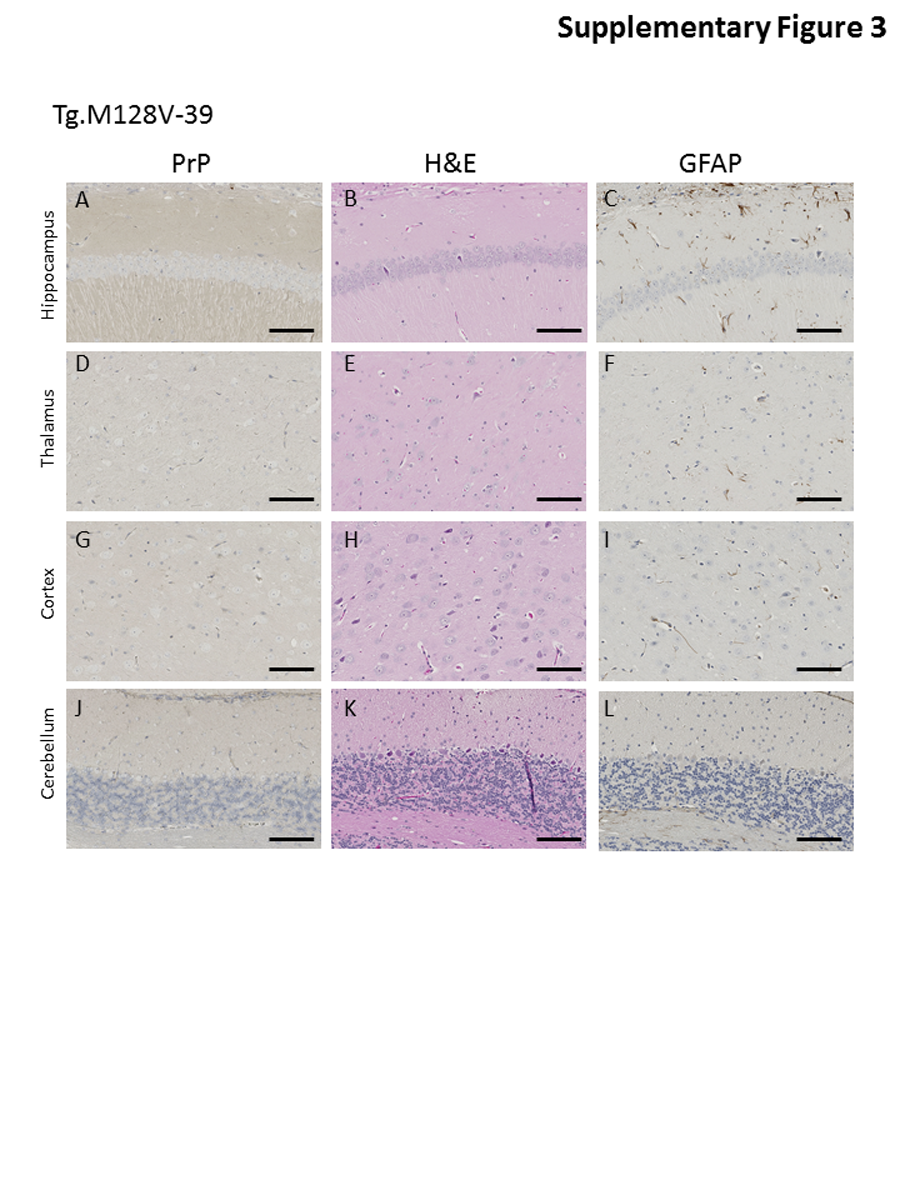

Supplement: S3 Fig — Photomicrographs of sagittal sections of healthy, aged Tg.M128V-39 mouse (576d). Immunostaining for PrP was performed after treatment of the slices with formic acid, slices destined for examination with H&E or GFAP were left untreated. The hippocampus (A-C), thalamus (D-F), cortex (G-I) and cerebellum (J-L) are shown. Vacuolation, PrPres and GFAP immunostaining are absent in all regions in the brains of these mice. Scale bar = 100 μm. (TIF) [file ppat.1006826.s003.TIF]

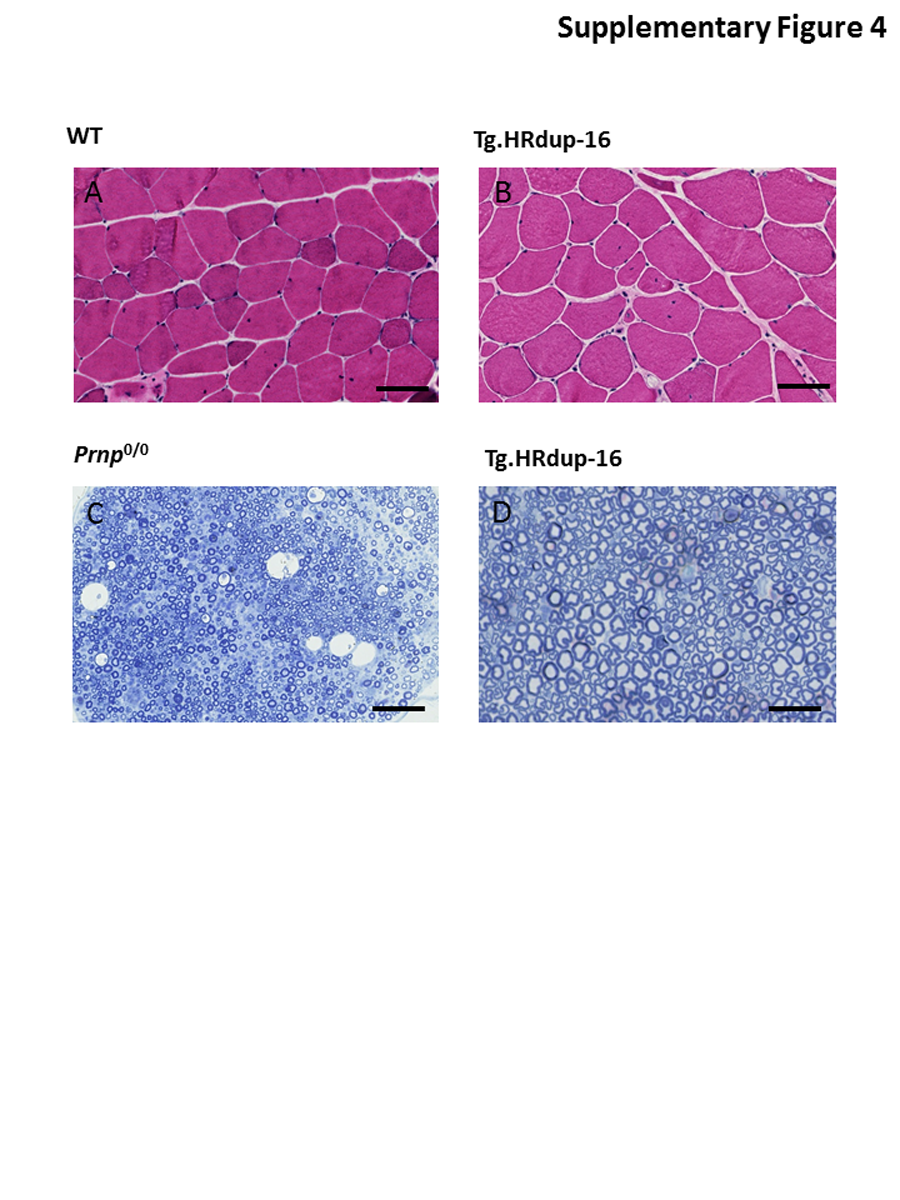

Supplement: S4 Fig — H&E stain of cross-section of quadriceps from aged WT (211d, A) and a Tg.HRdup-26 mouse (437 d, B). Scale bars 50 μm. Toluidine blue staining of a cross section of sciatic nerve from an aged Prnp0/0 (446d, C) showing irregular fibre diameters. A sick Tg.HRdup-26 mouse (437d, D). Scale bar = 25 μm. (TIF) [file ppat.1006826.s004.TIF]

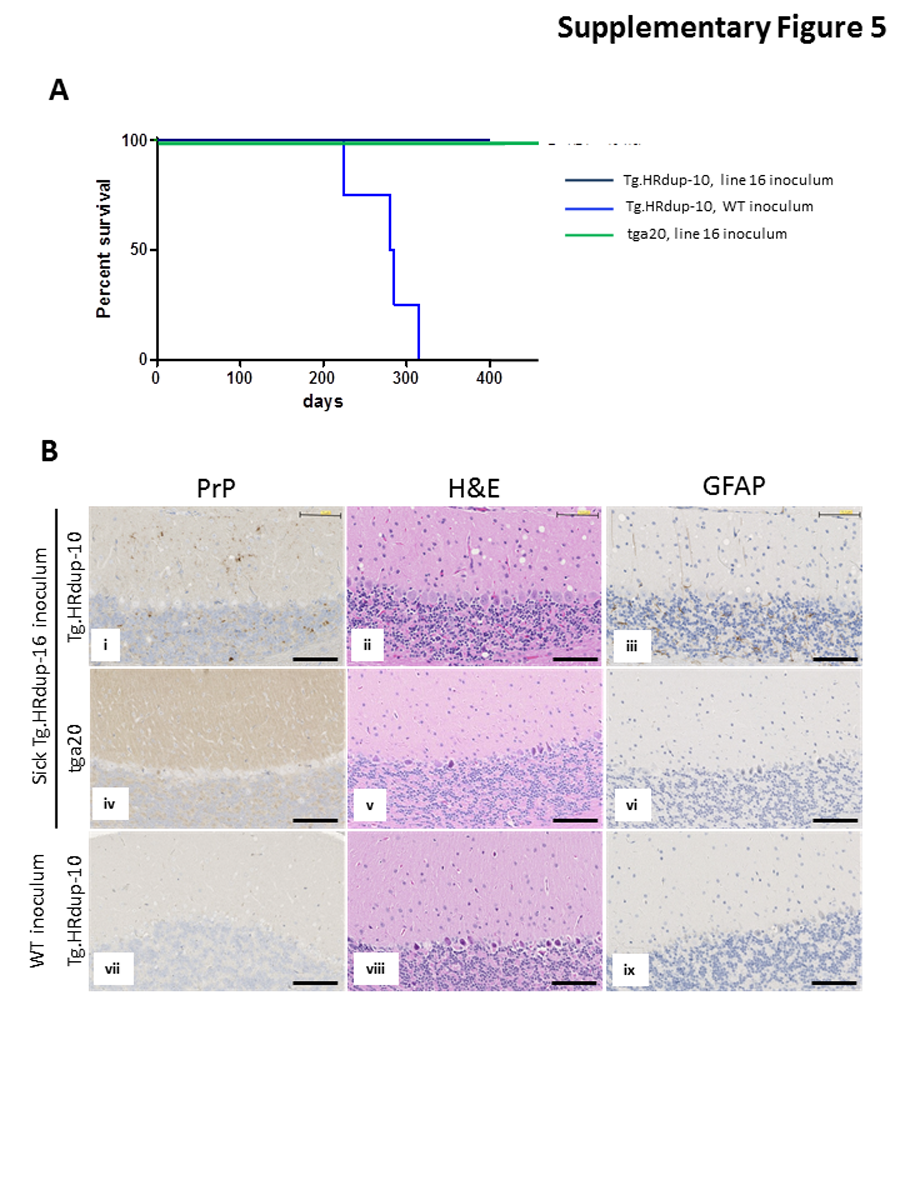

Supplement: S5 Fig — Low expressing Tg.HRdup-10 animals and tga20 (overexpressing WT PrP ~6-7x) animals received an intracranial inoculation with brain homogenate from a sick Tg.HRdup-26 animal or a healthy WT animal. The onset of clinical disease and pathological change was accelerated in the animals receiving the HRdup brain homogenate. A) Kaplan-Meier death curve for animals in the study. n = 4 for all groups. tga20 animals, with the exception of one that had to be euthanized due to intercurrent illness, were euthanized after 460 days. Tg.HRdup-10 animals inoculated with healthy brain homogenate were euthanized after 400 days. Tg.HRdup-10 animals inoculated with clinical Tg.HRdup-26 brain homogenate succumbed to disease at 276±38 days. B) Immunohistochemistry of the cerebellum of a Tg.HRdup-10 (top row) and tga20 animal (middle row) inoculated with the brain homogenate from a sick Tg.HRdup-26 animal or Tg.HRdup-10 animal inoculated material from a healthy WT animal (bottom row). i, iv, vii, PrPSc immunoreactivity with SAF83; ii, v, viii, H&E stain; iii, vi, ix: GFAP immunoreactivity. Scale bar = 75 μm. Note that only animals that succumbed to illness (and harboring protease resistant PrP) display PrP immunoreactivity, vacuolation and astrocytic gliosis associated with prion disease. (TIF) [file ppat.1006826.s005.TIF]

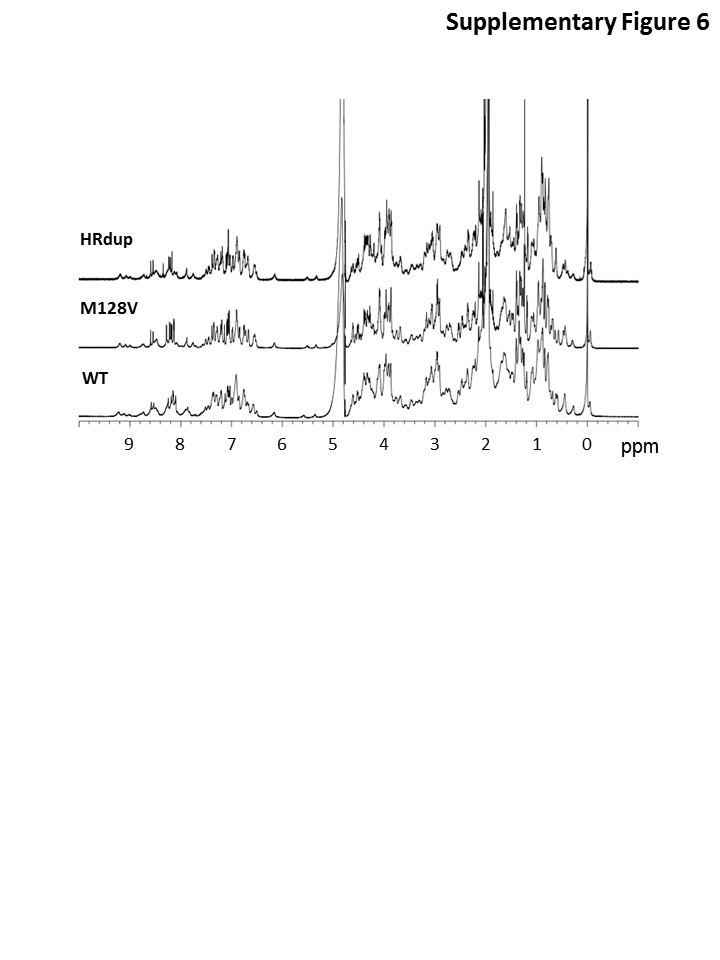

Supplement: S6 Fig — 1D 1H NMR full spectra are presented for HRdup, M128V and WT PrP (top to bottom). (TIF) [file ppat.1006826.s006.TIF]

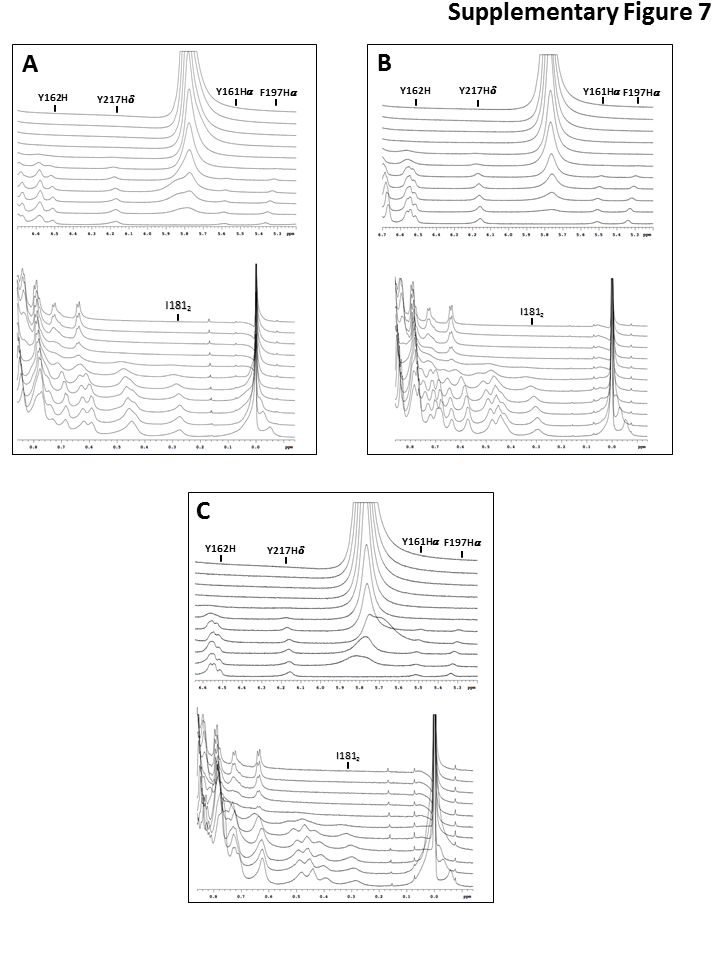

Supplement: S7 Fig — 1D 1H NMR experiment of WT (A), M128V (B) and HRdup PrP (C) titrated with urea from 0 to 10M. Upper panels show stacked plots of the aromatic region of spectra, ranging from 5.2 to 6.7ppm (resonances of Y162Hε, Y161Hα, F197Hα and Y217Hδ are indicated) while lower panels show stacked plots of the methyl region of spectra, ranging from -0.14 to 0.86 ppm (resonance of I181Hγ2 is indicated). (TIF) [file ppat.1006826.s007.TIF]

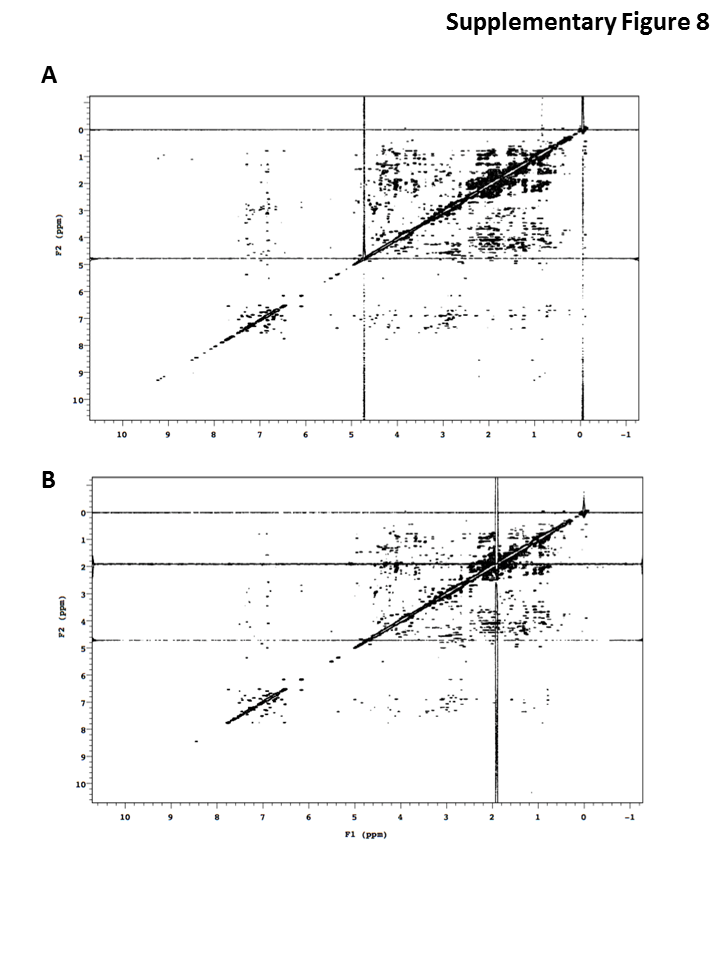

Supplement: S8 Fig — Full spectra of 2D 1H-1H NOESY of HRdup (A) and M128V PrP (B) are presented. (TIF) [file ppat.1006826.s008.TIF]

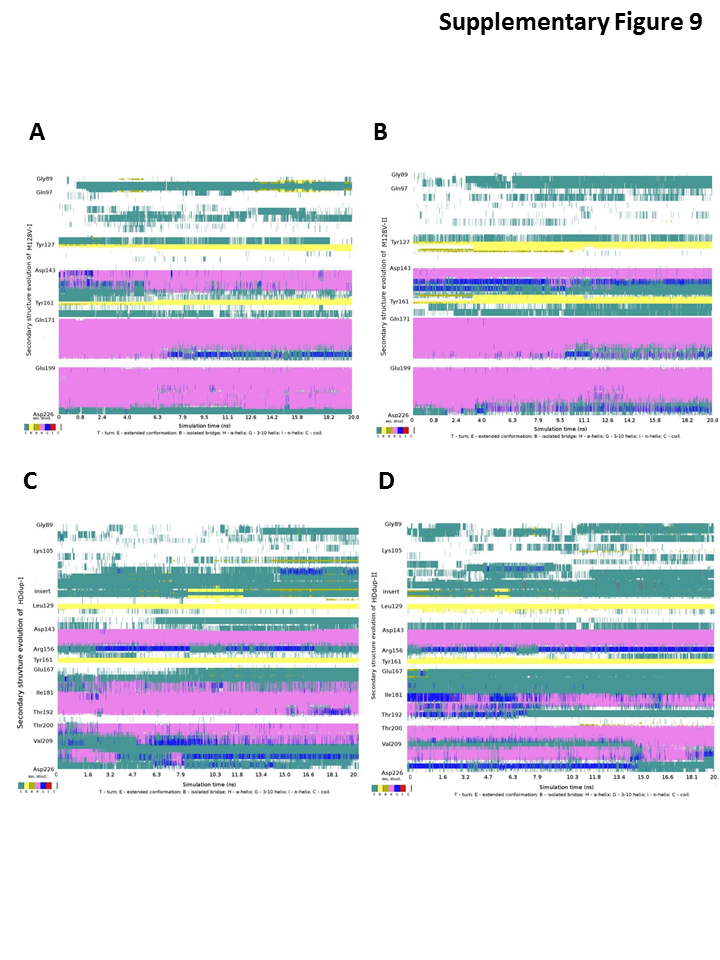

Supplement: S9 Fig — Shown are data from the M128V-I system (A), M128V-II system (B), HRdup-I system (C), and HRdup-II system (D). The total simulation time was 20 ns. Along the vertical axes, N-terminals are on the top and C-terminals are on the bottom. The legends underneath indicate the structure-color associations: T–turn (cyan), E–extended β conformation (yellow), B–isolated β bridge (dark yellow), H–α-helix (purple), G– 3-10-helix (blue), I–π-helix (red), and C–coil (white). The figures were obtained with the VMD package (43). (TIF) [file ppat.1006826.s009.TIF]

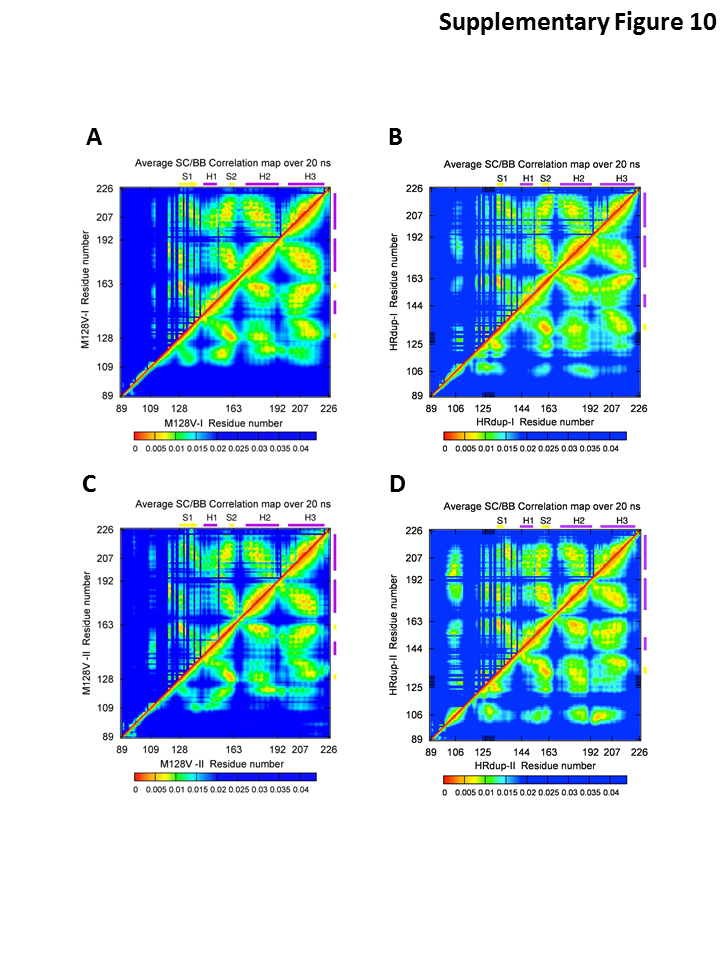

Supplement: S10 Fig — These are calculated for side-chain atoms (above the diagonal) and main-chain atoms (below the diagonal) in M128V-I system (A), HRdup-I system (B), M128V-II system (C), and HRdup-II system (D). High levels of correlations are indicated with red and yellow, medium levels are indicated with green and light-blue; and low levels with dark blue. Some of the dark blue lines in the side chain correlation maps correspond to glycine (no side chains) and alanine (short side chains) residues, which were excluded from the side-chain analysis. (TIF) [file ppat.1006826.s010.TIF]

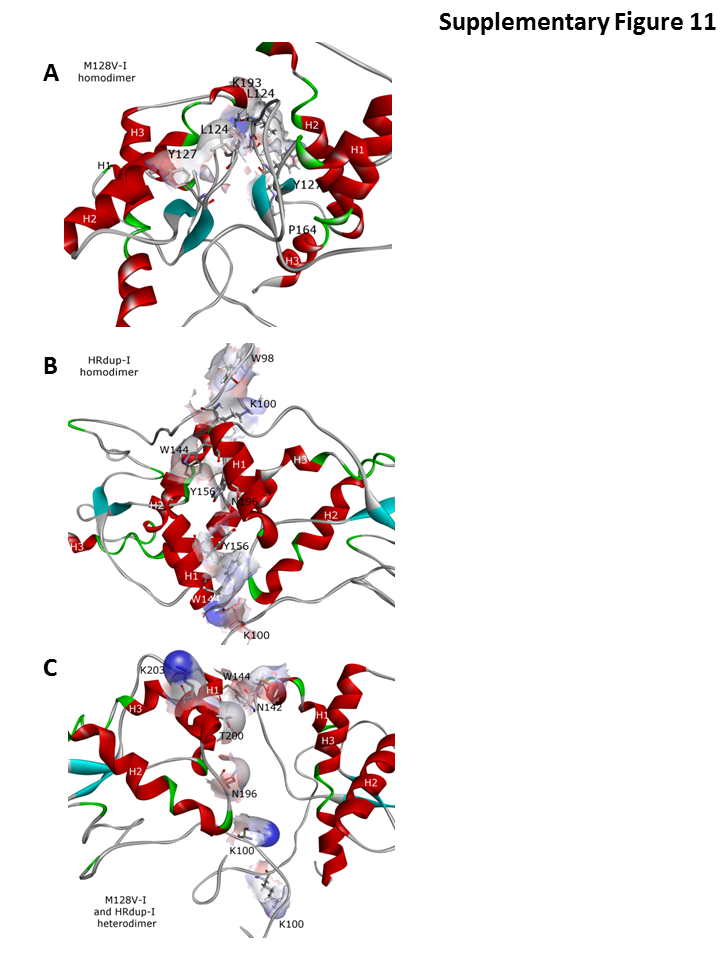

Supplement: S11 Fig — Close-up of predicted homodimers M128V-I (A) and HRdup-I (B), and heterodimer M128V-I HRdup-I (C). The residues on the binding interface are shown with lines and translucent surfaces colored according to the electrostatic charge (translucent pink–negative charge; translucent blue–positive charge; white–neutral). In the display of the first chain in the dimers, α-helices are indicated with opaque red color; β-strands with light blue; random coils with grey; and turns or helix disruptions with green. (TIF) [file ppat.1006826.s011.TIF]

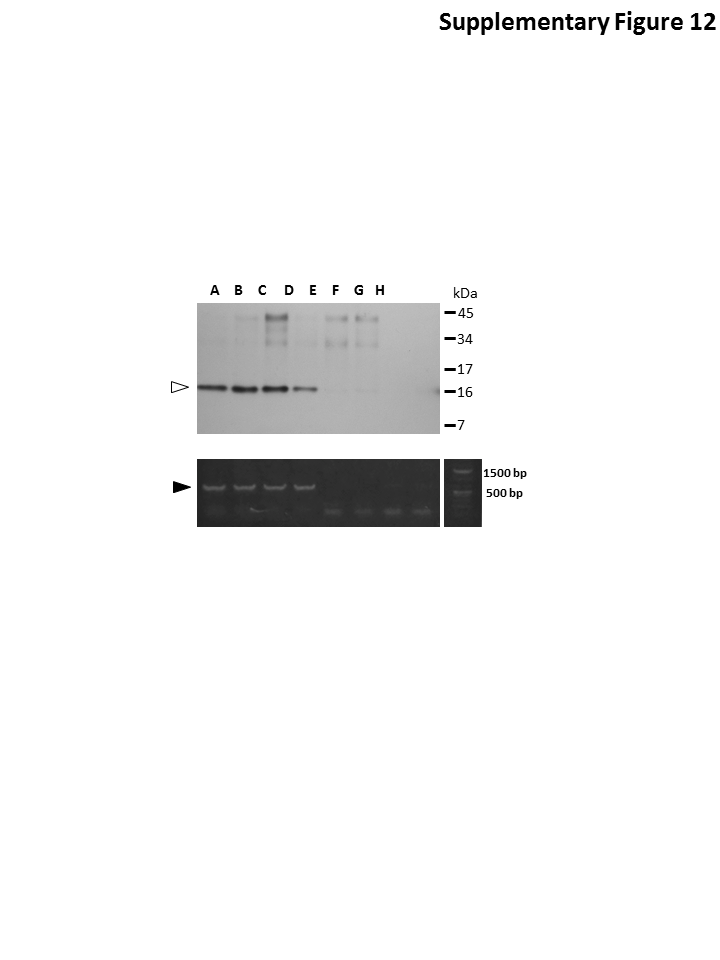

Supplement: S12 Fig — Upper panel, western blot analysis of brains of eight neonatal animals (A-H) from the Tg.HRdup-26 line. Antibody is Sha31. The four left-hand samples have the thermolysin-resistant species. Lower panel, corresponding PCR analyses of genomic DNA from eight neonatal mice with reaction products displayed on SYBR safe stained agarose gel. The diagnostic fragment for the PrP transgene migrates at 550 bp. (TIF) [file ppat.1006826.s012.TIF]
