# Supplementary material for: A novel Gerstmann-Sträussler-Scheinker disease mutation defines a precursor for amyloidogenic 8 kDa PrP fragments and reveals N-terminal structural changes shared by other GSS alleles
Source: PLoS Pathog. 2018 Jan 16;14(1):e1006826. doi: 10.1371/journal.ppat.1006826 (PMC5786331; doi:10.1371/journal.ppat.1006826)
Supplement: S1 Text — (DOCX) [file ppat.1006826.s013.docx]

**A novel Gerstmann-Sträussler-Scheinker disease mutation defines a precursor for amyloidogenic 8 kDa PrP fragments and reveals N-terminal structural changes shared by other GSS alleles.**

**SUPPLEMENTARY METHODS**

**Expression in mammalian cells**

pcDNA3.moPrP.wt was subjected to site directed mutagenesis to create pcDNA3.moPrP128V and pcDNA3.moPrPHRdup128V using the Agilent QuikChange II XL kit. 128V primers: SW132 5’-ggggggccttggtggctacgtgctggggagcgccatgagc-3’, SW133 5’-gctcatggcgctccccagcacgtagccaccaaggcccccc-3’. The sequences were then verified using the universal T7 and SP6 primers before excision and religation into pcDNA3 that had not been subjected to site directed mutagenesis. Rabbit kidney epithelial (RK13) cells were maintained in Dulbecco’s Modified Eagles Medium (DMEM; Invitrogen) that had been supplemented with 10% fetal bovine serum (Invitrogen) and 1% penicillin/streptomycin (Invitrogen) at 37°C and 5% CO2. Cells were transfected at a confluence of 80-90% using Lipofectamine 2000 (Invitrogen) according to the manufacturer's instructions.

**Immunocytochemistry**

RK13 cells were transfected with the indicated pcDNA3 PrP vector. 24 h after transfection, cells were re-plated on glass coverslips and given 24 h to recover. After rinsing twice with PBS and fixation with 4% paraformaldehyde, cells were washed 3 times with PBS before permeabilization with 0.2% Triton X-100 (Sigma) or were left un-permeabilized. Cells were rinsed 3 times with PBS and incubated in Sha31 (1:5000) overnight at 4 °C with rocking. Following 3 PBS washes, cells were blocked with 2% goat serum (Invitrogen) and incubated with goat α-mouse Alexa Fluor 488 (Invitrogen, 1:300) for 2 h at room temperature. Nuclei were stained with 1 μg/mL Hoechst and visualized using a Eclipse 90I microscope (Nikon) and a CFI PL 40X/ N.A. 0.75 lens (Nikon) using the following excitation/emission filter properties: 325 – 375/500 – 575 nm with a 495 nm long-pass filter (blue channel) and 440-510/475-575 nm with a 495 nm long-pass filter (green channel). Images were acquired at room temperature with a Retiga 2000R mono cooled camera, fast 1394 using NIS-Elements AR advanced research software.

**Cell Surface Biotinylation Assay**

The Pierce cell surface protein isolation kit was scaled down to a 6-well format and performed according to manufacturer's instructions.
