# Supplementary material for: A novel Gerstmann-Sträussler-Scheinker disease mutation defines a precursor for amyloidogenic 8 kDa PrP fragments and reveals N-terminal structural changes shared by other GSS alleles
Source: PLoS Pathog. 2018 Jan 16;14(1):e1006826. doi: 10.1371/journal.ppat.1006826 (PMC5786331; doi:10.1371/journal.ppat.1006826)
Supplement: S1 Table — (DOCX) [file ppat.1006826.s014.docx]

**Table S1. Pathology in mouse Tg.HRdup lines**

| **Brain region** | | **Spongiosis** | | | **PrP^D^** | **Tau** | **Amyloid** |
| --- | --- | --- | --- | --- | --- | --- | --- |
| Cortex | | Line 10: 3 Mi, 1 Mo, 3 Neg; Line 26: 10 Mi, 5 Mo, 3 St, 2 Neg; Line 32: 6 Mi, 1 Mo) | | | Focal deposit (0/7 line 10; 0/1 line 13; 8/20 line 26; 0/7 line 32) | None (0/1 line 13; 0/4 line 26; 0/1 line 32) | None (0/1 line 13; 0/3 line 26; 0/3 line 32) |
| White matter | | Line 10: 4 Mi, 1 Mo, 2 Neg; Line 26: 6 Mi, 6 Mo, 2 St, 6 Neg; Line 32: 3 Mi, 4 Mo) | | | Focal deposit (1/7 line 10; 0/1 line 13; 9/20 line 26; 0/7 line 32) | None (0/1 line 13; 0/4 line 26; 0/1 line 32) | None (0/1 line 13; 0/3 line 26; 0/3 line 32) |
| Hippocampus | | Line 10: 1 Mi, 3 Mo, 1St, 2 Neg; Line 26: 4 Mi, 6 Mo, 6 St, 4 Neg; Line 32: 6 Mi, 1 Mo) | | | Focal deposit (3/7 line 10; 0/1 line 13; 9/20 line 26; 0/7 line 32) | None (0/1 line 13; 0/4 line 26; 0/1 line 32) | None (0/1 line 13; 0/3 line 26; 0/3 line 32) |
| Striatum | | Line 10: 1 Mi, 5 Mo, 1 Neg; Line 26: 6 Mi, 4 Mo, 4 St, 6 Neg; Line 32: 4 Mi, 2 Mo, 1 Neg) | | | Focal deposit (0/7 line 10; 0/1 line 13; 8/20 line 26; 0/7 line 32) | None ('0/1 line 13; 0/4 line 26; 0/1 line 32) | None (0/1 line 13; 0/3 line 26; 0/3 line 32) |
| Thalamus | | Line 10: 1 Mi, 5 Mo, 1 Neg; Line 26: 6 Mi, 9 Mo, 4 St, 1 Neg; Line 32: 2 Mi, 5 Mo) | | | Focal deposit (0/7 line 10; 0/1 line 13; 8/20 line 26; 0/7 line 32) | None ('0/1 line 13; 0/4 line 26; 0/1 line 32) | None (0/1 line 13; 0/3 line 26; 0/3 line 32) |
| Brainstem | | Line 10: 3 Mi, 1 Mo, 3 Neg; Line 26: 8 Mi, 6 Mo, 0 St, 6 Neg; Line 32: 1 Mi, 6 Mo) | | | Focal deposit (3/7 line 10; 0/1 line 13; 7/20 line 26; 0/7 line 32) | None ('0/1 line 13; 0/4 line 26; 0/1 line 32) | None (0/1 line 13; 0/3 line 26; 0/3 line 32) |
| Cerebellum | | Line 10: 1 Mi, 1 Mo, 3 St, 2 Neg; Line 26: 10 Mi, 5 Mo, 4 St, 1 Neg; Line 32: 1 Mi, 4 Mo, 2 St) | | | Focal deposit (3/7 line 10; 0/1 line 13; 8/20 line 26; 0/7 line 32) | None ('0/1 line 13; 0/4 line 26; 0/1 line 32) | None (0/1 line 13; 0/3 line 26; 0/3 line 32) |
|  |  | |  |  |  |  |  |

Line 32 age range examined is 149-237d, Line 26 age range 111-482d, line 10 age range 308-636d

Mi = Mild, Mo = moderate, St = strong and neg = negative
