# Supplementary material for: A novel Gerstmann-Sträussler-Scheinker disease mutation defines a precursor for amyloidogenic 8 kDa PrP fragments and reveals N-terminal structural changes shared by other GSS alleles
Source: PLoS Pathog. 2018 Jan 16;14(1):e1006826. doi: 10.1371/journal.ppat.1006826 (PMC5786331; doi:10.1371/journal.ppat.1006826)
Supplement: S2 Table — (DOCX) [file ppat.1006826.s015.docx]

**Table S2. *m* values Table for 5 PrP residues in a urea denaturation experiment**

| **m-Value**  **(kJ mol^-1^ M^-1^)** | **WT** | **M128V** | **HRdup** |
| --- | --- | --- | --- |
| I181Hγ2 | 3.2±0.5 | 3.8±0.6 | 6.8±2.1 |
| Y162H$\varepsilon$ | 2.9±0.5 | 3.9±0.4 | 5.1±0.9 |
| Y161Hα | 4.1±0.9 | 6.5±2.6 | 5.5±0.6 |
| F197Hα | 7.0±1.3 | 5.0±0.5 | 4.1±1.0 |
| Y217Hα | 2.9±0.4 | 3.6±0.3 | 4.6±1.6 |
