# Supplementary material for: A novel Gerstmann-Sträussler-Scheinker disease mutation defines a precursor for amyloidogenic 8 kDa PrP fragments and reveals N-terminal structural changes shared by other GSS alleles
Source: PLoS Pathog. 2018 Jan 16;14(1):e1006826. doi: 10.1371/journal.ppat.1006826 (PMC5786331; doi:10.1371/journal.ppat.1006826)
Supplement: S3 Table — (DOCX) [file ppat.1006826.s016.docx]

**Supplementary Table S3: The results of SWISS-MODEL template library search with BLAST and HHBlits**

The search was for evolutionary related structures matching the target HRdup construct with the M128V substitution and LGGLGGYV insert sequence. For GMQE and QMEAN4 scores description, see Methods above.

|  | **Template (PDB ID. model#. chain ID)** | **Sequence Identity, %** | **Oligo-state** | **Found by** | **Method** | **Resolution** | **Sequence Similarity** | **Range (WT)** | **Coverage** | **Description** | **GMQE** | **QMEAN4** |
| --- | --- | --- | --- | --- | --- | --- | --- | --- | --- | --- | --- | --- |
| **HRdup models** | [4kml.1.A](file:///C:\Users\doroshl\AppData\Local\Temp\_tc\Claudia_MoPrP_insert_2015-06-07\model\03\templates\4kml.1.A.pdb) | 90.34 | hetero-oligomer | HHblits | X-ray | 1.50Å | 0.62 | 116 - 224 | 0.95 | Major prion protein | 0.50 | -0.97 |
|  | [1qlz.1.A](file:///C:\Users\doroshl\AppData\Local\Temp\_tc\Claudia_MoPrP_insert_2015-06-07\model\02\templates\1qlz.1.A.pdb) | 90.34 | monomer | HHblits | NMR | NA | 0.62 | 114 - 227 | 0.95 | PRION PROTEIN | 0.49 | -1.32 |
|  | [4ma7.1.A](file:///C:\Users\doroshl\AppData\Local\Temp\_tc\Claudia_MoPrP_insert_2015-06-07\model\14\templates\4ma7.1.A.pdb) | 99.12 | hetero-oligomer | HHblits | X-ray | 1.97Å | 0.63 | 118 - 227 | 0.53 | Major prion protein | 0.46 | -1.56 |
|  | [4ma8.1.A](file:///C:\Users\doroshl\AppData\Local\Temp\_tc\Claudia_MoPrP_insert_2015-06-07\model\13\templates\4ma8.1.A.pdb) | 99.12 | hetero-oligomer | HHblits | X-ray | 2.20Å | 0.63 | 118 - 225 | 0.53 | Major prion protein | 0.45 | -1.49 |
|  | [2l1h.1.A](file:///C:\Users\doroshl\AppData\Local\Temp\_tc\Claudia_MoPrP_insert_2015-06-07\model\16\templates\2l1h.1.A.pdb) | 99.11 | monomer | HHblits | NMR | NA | 0.63 | 123 - 226 | 0.52 | Major prion protein | 0.45 | -1.94 |
|  | [1b10.1.A](file:///C:\Users\doroshl\AppData\Local\Temp\_tc\Claudia_MoPrP_insert_2015-06-07\model\06\templates\1b10.1.A.pdb) | 93.62 | monomer | HHblits | NMR | NA | 0.62 | 124 - 227 | 0.65 | PRION PROTEIN | 0.43 | -3.25 |
|  | [2ku5.1.A](file:///C:\Users\doroshl\AppData\Local\Temp\_tc\Claudia_MoPrP_insert_2015-06-07\model\19\templates\2ku5.1.A.pdb) | 98.21 | monomer | HHblits | NMR | NA | 0.62 | 120 - 231 | 0.52 | Major prion protein | 0.43 | -3.13 |
|  | [**2l39**.1.A](file:///C:\Users\doroshl\AppData\Local\Temp\_tc\Claudia_MoPrP_insert_2015-06-07\model\15\templates\2l39.1.A.pdb) | 98.25 | monomer | HHblits | NMR | NA | 0.63 | 118 - 231 | 0.53 | Major prion protein | 0.41 | -4.03 |
