# Supplementary material for: A novel Gerstmann-Sträussler-Scheinker disease mutation defines a precursor for amyloidogenic 8 kDa PrP fragments and reveals N-terminal structural changes shared by other GSS alleles
Source: PLoS Pathog. 2018 Jan 16;14(1):e1006826. doi: 10.1371/journal.ppat.1006826 (PMC5786331; doi:10.1371/journal.ppat.1006826)
Supplement: S4 Table — (DOCX) [file ppat.1006826.s017.docx]

**Supplementary Table S4: Time-averaged β-content occupancies in four MD trajectories**

| **Residue** | **M128V-I** | **M128V-II** | **HRdup-I** | **HRdup-II** |
| --- | --- | --- | --- | --- |
| **Q90** | 6.2% |  |  |  |
| **G91** | 12.6% |  |  |  |
| **G92** | 11.4% |  |  |  |
| **N96** | 11.7% |  |  |  |
| **Q97** | 17.8% |  |  |  |
| **K105** |  |  |  | 10.0% |
| **H110** |  |  | 28.8% |  |
| **Y127** |  |  | 38.0% | 4.2% |
| **V128** | 99.6% | 100.0% | 18.5% | 20.3% |
| **ins-G3** |  |  | 18.4% | 20.4% |
| **ins-L4** |  |  | 12.2% | 4.0% |
| **ins-G5** |  |  | 6.0% |  |
| **ins-V8** |  |  | 79.1% | 100.0% |
| **L129** | 92.2% | 92.8% | 79.1% | 100.0% |
| **G130** | 67.0% | 78.0% | 78.7% | 97.6% |
| **S131** | 19.3% | 68.6% |  | 27.6% |
| **A132** |  | 48.9% |  | 22.8% |
| **M133** |  | 34.4% |  |  |
| **N158** |  | 50.9% |  |  |
| **Q159** | 19.3% | 68.6% |  | 22.9% |
| **V160** | 67.0% | 78.0% | 78.6% | 97.6% |
| **Y161** | 92.2% | 92.8% | 78.9% | 100.0% |
| **Y162** | 99.6% | 100.0% | 78.9% | 100.0% |
| **D166** |  |  | 3.9% | 4.4% |
| **N170** |  |  | 3.8% | 4.1% |
| **T198** |  |  |  | 10.0% |
