# Supplementary material for: A novel Gerstmann-Sträussler-Scheinker disease mutation defines a precursor for amyloidogenic 8 kDa PrP fragments and reveals N-terminal structural changes shared by other GSS alleles
Source: PLoS Pathog. 2018 Jan 16;14(1):e1006826. doi: 10.1371/journal.ppat.1006826 (PMC5786331; doi:10.1371/journal.ppat.1006826)
Supplement: S5 Table — (DOCX) [file ppat.1006826.s018.docx]

**Table S5: Per residue solvent accessible surface area (SASA)**

Per residue solvent accessible surface area (SASA) was calculated from the last 2 ns of the four 20 ns trajectories. Difference in per-residue SASA for models M128V and HRDdup were averaged over two trajectories each; total per-residue SASA (11); and difference in weighted average per-residue SASA for models M128V and HRDdup. In the first two columns, hydrophobic residues are highlighted in blue, and charged residues are highlighted in red. In the last column, residues that exhibit more than 25% differences in SASA are highlighted in dark red.

| **Residue** | **SASA, nm^2^** | | | | **Difference in average SASA(^a^),**  **nm^2^** | **Maximal**  **SASA(^b^), nm^2^** | **Difference**  **in weighted average SASA, %(^c^)** |
| --- | --- | --- | --- | --- | --- | --- | --- |
|  | **M128V-I** | **M128V-II** | **HRdup-I** | **HRdup-II** |  |  |  |
| G89 | 0.921 | 0.989 | 0.878 | 0.806 | -0.113 | 0.813 | -13.89 |
| Q90 | 1.405 | 0.993 | 0.916 | 0.951 | -0.265 | 1.812 | -14.63 |
| G91 | 0.282 | 0.330 | 0.569 | 0.626 | 0.292 | 0.813 | 35.92 |
| G92 | 0.554 | 0.249 | 0.785 | 0.534 | 0.258 | 0.813 | 31.69 |
| G93 | 0.602 | 0.603 | 0.738 | 0.745 | 0.139 | 0.813 | 17.09 |
| T94 | 1.387 | 0.555 | 0.699 | 1.002 | -0.120 | 1.401 | -8.59 |
| H95 | 1.282 | 1.752 | 1.224 | 1.330 | -0.240 | 1.845 | -13.02 |
| N96 | 0.850 | 0.856 | 1.073 | 0.641 | 0.004 | 1.566 | 0.23 |
| Q97 | 1.136 | 0.479 | 1.127 | 1.232 | 0.372 | 1.819 | 20.45 |
| W98 | 1.318 | 1.074 | 1.321 | 2.012 | 0.470 | 2.538 | 18.53 |
| N99 | 1.172 | 0.946 | 1.255 | 0.743 | -0.060 | 1.566 | -3.82 |
| K100 | 1.416 | 1.489 | 1.161 | 1.578 | -0.083 | 2.049 | -4.03 |
| P101 | 0.496 | 0.901 | 0.747 | 0.769 | 0.060 | 1.364 | 4.37 |
| S102 | 0.843 | 1.079 | 0.920 | 0.672 | -0.165 | 1.159 | -14.23 |
| K103 | 1.280 | 1.566 | 1.469 | 1.521 | 0.072 | 2.049 | 3.51 |
| P104 | 0.784 | 1.172 | 0.675 | 0.540 | -0.371 | 1.364 | -27.18 |
| K105 | 1.729 | 1.730 | 1.409 | 1.418 | -0.316 | 2.049 | -15.40 |
| T106 | 1.152 | 0.859 | 0.791 | 0.292 | -0.464 | 1.401 | -33.12 |
| N107 | 0.992 | 1.270 | 0.523 | 1.053 | -0.343 | 1.566 | -21.87 |
| L108 | 1.659 | 0.798 | 0.609 | 0.790 | -0.529 | 1.821 | -29.07 |
| K109 | 1.378 | 1.294 | 1.074 | 0.889 | -0.354 | 2.049 | -17.29 |
| H110 | 0.993 | 1.465 | 0.962 | 1.334 | -0.081 | 1.845 | -4.37 |
| V111 | 0.572 | 0.369 | 0.975 | 0.863 | 0.448 | 1.527 | 29.36 |
| A112 | 0.342 | 0.727 | 0.470 | 0.549 | -0.025 | 1.085 | -2.29 |
| G113 | 0.455 | 0.611 | 0.758 | 0.514 | 0.103 | 0.813 | 12.64 |
| A114 | 0.554 | 0.373 | 0.625 | 0.443 | 0.071 | 1.085 | 6.51 |
| A115 | 0.319 | 0.775 | 0.961 | 0.949 | 0.408 | 1.085 | 37.58 |
| A116 | 0.424 | 0.748 | 0.847 | 0.585 | 0.130 | 1.085 | 11.98 |
| A117 | 0.756 | 0.600 | 0.345 | 0.898 | -0.057 | 1.085 | -5.24 |
| G118 | 0.334 | 0.417 | 0.458 | 0.578 | 0.142 | 0.813 | 17.49 |
| A119 | 0.321 | 0.491 | 0.528 | 0.290 | 0.003 | 1.085 | 0.28 |
| V120 | 0.331 | 0.299 | 1.224 | 0.877 | 0.735 | 1.527 | 48.16 |
| V121 | 1.099 | 0.707 | 0.443 | 1.094 | -0.134 | 1.527 | -8.77 |
| G122 | 0.340 | 0.403 | 0.123 | 0.544 | -0.038 | 0.813 | -4.69 |
| G123 | 0.699 | 0.609 | 0.070 | 0.316 | -0.461 | 0.813 | -56.71 |
| L124 | 0.911 | 0.691 | 0.556 | 0.648 | -0.199 | 1.821 | -10.92 |
| G125 | 0.645 | 0.641 | 0.400 | 0.626 | -0.130 | 0.813 | -16.00 |
| G126 | 0.565 | 0.559 | 0.155 | 0.112 | -0.428 | 0.813 | -52.67 |
| Y127 | 0.717 | 0.646 | 0.868 | 0.633 | 0.069 | 2.167 | 3.19 |
| V128 | 0.621 | 0.617 | 0.016 | 0.308 | -0.457 | 1.527 | -29.94 |
| insL1 | x | x | 0.443 | 1.108 | x | 1.821 | x |
| insG2 | x | x | 0.424 | 0.122 | x | 0.813 | x |
| insG3 | x | x | 0.169 | 0.073 | x | 0.813 | x |
| insL4 | x | x | 1.107 | 0.744 | x | 1.821 | x |
| insG5 | x | x | 0.408 | 0.214 | x | 0.813 | x |
| insG6 | x | x | 0.489 | 0.449 | x | 0.813 | x |
| insY7 | x | x | 0.330 | 0.452 | x | 2.167 | x |
| insV8 | x | x | 0.405 | 0.538 | x | 1.527 | x |
| L129 | 0.951 | 0.843 | 0.451 | 0.843 | -0.164 | 1.821 | -8.99 |
| G130 | 0.129 | 0.106 | 0.229 | 0.072 | -0.128 | 0.813 | -15.75 |
| S131 | 0.514 | 0.729 | 0.668 | 0.696 | -0.005 | 1.159 | -0.46 |
| A132 | 0.189 | 0.405 | 0.773 | 0.628 | 0.262 | 1.085 | 24.17 |
| M133 | 1.176 | 1.306 | 0.641 | 0.437 | -0.314 | 1.991 | -15.79 |
| S134 | 0.740 | 0.571 | 0.525 | 0.750 | 0.023 | 1.159 | 2.02 |
| R135 | 1.749 | 1.108 | 1.622 | 0.803 | -0.039 | 2.464 | -1.57 |
| P136 | 0.647 | 0.723 | 0.355 | 0.261 | -0.418 | 1.364 | -30.66 |
| M137 | 1.500 | 0.670 | 1.763 | 1.315 | 0.608 | 1.991 | 30.53 |
| I138 | 0.300 | 0.429 | 1.176 | 0.624 | 0.360 | 1.815 | 19.82 |
| H139 | 1.378 | 0.860 | 0.449 | 1.054 | -0.618 | 1.845 | -33.52 |
| F140 | 0.518 | 0.688 | 0.515 | 0.701 | -0.019 | 2.085 | -0.91 |
| G141 | 0.617 | 0.531 | 0.392 | 0.560 | 0.020 | 0.813 | 2.46 |
| N142 | 0.793 | 0.487 | 1.037 | 0.771 | -0.083 | 1.566 | -5.31 |
| D143 | 0.752 | 0.754 | 0.781 | 0.758 | -0.156 | 1.462 | -10.65 |
| W144 | 0.843 | 0.805 | 1.568 | 1.406 | 0.992 | 2.538 | 39.09 |
| E145 | 0.643 | 0.362 | 0.575 | 0.636 | 0.016 | 1.771 | 0.92 |
| D146 | 1.053 | 0.780 | 0.314 | 0.315 | -0.799 | 1.462 | -54.63 |
| R147 | 1.315 | 1.362 | 1.118 | 1.161 | 0.198 | 2.464 | 8.03 |
| Y148 | 0.613 | 0.737 | 0.993 | 0.878 | 0.485 | 2.167 | 22.38 |
| Y149 | 0.305 | 0.296 | 0.383 | 0.113 | -0.168 | 2.167 | -7.75 |
| R150 | 1.100 | 1.181 | 1.286 | 1.155 | 0.548 | 2.464 | 22.24 |
| E151 | 1.167 | 1.099 | 1.236 | 1.216 | 0.270 | 1.771 | 15.26 |
| N152 | 0.182 | 0.146 | 0.255 | 0.263 | 0.151 | 1.566 | 9.64 |
| M153 | 0.511 | 0.536 | 0.756 | 0.506 | 0.254 | 1.991 | 12.75 |
| Y154 | 1.179 | 1.173 | 1.529 | 1.542 | 0.690 | 2.167 | 31.84 |
| R155 | 0.756 | 0.569 | 0.580 | 0.402 | -0.173 | 2.464 | -7.01 |
| Y156 | 0.283 | 0.288 | 0.254 | 0.124 | -0.334 | 2.167 | -15.42 |
| P157 | 0.463 | 0.526 | 0.215 | 0.147 | -0.626 | 1.364 | -45.92 |
| N158 | 0.237 | 0.245 | 0.722 | 0.714 | 0.301 | 1.566 | 19.23 |
| Q159 | 0.637 | 0.745 | 0.572 | 0.871 | -0.369 | 1.819 | -20.26 |
| V160 | 0.025 | 0.034 | 0.098 | 0.078 | -0.869 | 1.527 | -56.91 |
| Y161 | 0.500 | 0.244 | 0.206 | 0.293 | -0.175 | 2.167 | -8.06 |
| Y162 | 0.389 | 0.513 | 0.248 | 0.686 | -0.204 | 2.167 | -9.44 |
| R163 | 0.745 | 0.571 | 0.737 | 1.248 | 0.355 | 2.464 | 14.43 |
| P164 | 0.678 | 0.762 | 0.212 | 0.743 | -0.381 | 1.364 | -27.90 |
| V165 | 1.019 | 1.152 | 0.407 | 1.181 | -0.101 | 1.527 | -6.59 |
| D166 | 1.188 | 0.597 | 0.473 | 0.674 | -0.143 | 1.462 | -9.78 |
| Q167 | 1.101 | 1.544 | 1.131 | 1.387 | 0.614 | 1.819 | 33.74 |
| Y168 | 1.412 | 1.889 | 1.808 | 1.824 | 0.466 | 2.167 | 21.49 |
| S169 | 0.861 | 0.348 | 0.747 | 0.597 | 0.026 | 1.159 | 2.27 |
| N170 | 0.537 | 0.955 | 0.609 | 0.813 | 0.421 | 1.566 | 26.87 |
| Q171 | 0.890 | 0.528 | 0.886 | 0.699 | 0.250 | 1.819 | 13.74 |
| N172 | 1.044 | 1.038 | 0.799 | 0.824 | -0.103 | 1.566 | -6.57 |
| N173 | 0.561 | 0.770 | 1.115 | 0.878 | 0.566 | 1.566 | 36.15 |
| F174 | 0.505 | 0.245 | 0.191 | 0.661 | 0.085 | 2.085 | 4.07 |
| V175 | 0.274 | 0.189 | 0.526 | 0.517 | 0.194 | 1.527 | 12.72 |
| H176 | 1.220 | 1.290 | 1.544 | 1.642 | 0.384 | 1.845 | 20.83 |
| D177 | 0.396 | 0.431 | 0.701 | 0.795 | 0.399 | 1.462 | 27.29 |
| C178 | 0.053 | 0.044 | 0.066 | 0.245 | -0.001 | 1.384 | -0.06 |
| V179 | 0.208 | 0.195 | 0.321 | 0.113 | -0.075 | 1.527 | -4.89 |
| N180 | 0.783 | 0.785 | 0.623 | 0.879 | -0.169 | 1.566 | -10.82 |
| I181 | 0.215 | 0.299 | 0.080 | 0.366 | -0.076 | 1.815 | -4.18 |
| T182 | 0.149 | 0.178 | 0.097 | 0.045 | -0.060 | 1.401 | -4.30 |
| I183 | 0.359 | 0.381 | 0.252 | 0.276 | -0.482 | 1.815 | -26.58 |
| K184 | 1.022 | 1.197 | 0.978 | 0.963 | -0.077 | 2.049 | -3.78 |
| Q185 | 0.172 | 0.303 | 0.099 | 0.119 | -0.492 | 1.819 | -27.03 |
| H186 | 0.188 | 0.260 | 0.363 | 0.505 | 0.031 | 1.845 | 1.71 |
| T187 | 0.286 | 0.375 | 0.423 | 0.235 | -0.062 | 1.401 | -4.43 |
| V188 | 1.041 | 1.057 | 0.613 | 0.532 | -0.395 | 1.527 | -25.87 |
| T189 | 0.350 | 0.382 | 0.362 | 0.597 | -0.173 | 1.401 | -12.38 |
| T190 | 0.198 | 0.113 | 0.177 | 0.831 | 0.030 | 1.401 | 2.15 |
| T191 | 1.156 | 1.134 | 0.809 | 1.172 | -0.178 | 1.401 | -12.71 |
| T192 | 0.944 | 1.074 | 0.519 | 1.237 | 0.175 | 1.401 | 12.51 |
| K193 | 1.415 | 1.029 | 0.876 | 1.365 | 0.267 | 2.049 | 13.01 |
| G194 | 0.546 | 0.617 | 0.548 | 0.378 | -0.048 | 0.813 | -5.94 |
| E195 | 0.588 | 0.496 | 0.614 | 0.515 | -0.205 | 1.771 | -11.58 |
| N196 | 1.122 | 0.895 | 1.035 | 0.460 | 0.105 | 1.566 | 6.71 |
| F197 | 0.906 | 0.956 | 0.172 | 0.127 | -0.389 | 2.085 | -18.65 |
| T198 | 0.704 | 0.750 | 0.720 | 0.552 | -0.060 | 1.401 | -4.28 |
| E199 | 1.258 | 1.180 | 1.196 | 1.047 | 0.145 | 1.771 | 8.21 |
| T200 | 0.583 | 0.461 | 1.045 | 0.921 | 0.596 | 1.401 | 42.53 |
| D201 | 0.457 | 0.293 | 0.373 | 0.124 | -0.015 | 1.462 | -1.00 |
| V202 | 0.440 | 0.476 | 0.439 | 0.217 | -0.209 | 1.527 | -13.69 |
| K203 | 1.154 | 0.951 | 1.427 | 1.397 | 0.582 | 2.049 | 28.41 |
| M204 | 0.085 | 0.162 | 1.008 | 0.254 | 0.502 | 1.991 | 25.19 |
| M205 | 0.152 | 0.170 | 0.070 | 0.045 | -0.021 | 1.991 | -1.05 |
| E206 | 0.755 | 0.688 | 0.828 | 0.693 | 0.147 | 1.771 | 8.32 |
| R207 | 0.992 | 0.693 | 0.720 | 0.995 | 0.249 | 2.464 | 10.10 |
| V208 | 0.293 | 0.192 | 0.131 | 0.137 | -0.183 | 1.527 | -11.97 |
| V209 | 0.093 | 0.069 | 0.267 | 0.169 | -0.140 | 1.527 | -9.17 |
| E210 | 0.700 | 0.634 | 0.933 | 0.858 | 0.424 | 1.771 | 23.96 |
| Q211 | 0.725 | 0.505 | 0.393 | 0.654 | -0.523 | 1.819 | -28.77 |
| M212 | 0.281 | 0.174 | 0.075 | 0.117 | -0.677 | 1.991 | -33.97 |
| C213 | 0.012 | 0.005 | 0.024 | 0.100 | 0.029 | 1.384 | 2.08 |
| V214 | 0.657 | 0.472 | 0.515 | 0.563 | 0.041 | 1.527 | 2.70 |
| T215 | 0.461 | 0.225 | 0.583 | 0.587 | -0.054 | 1.401 | -3.84 |
| Q216 | 0.098 | 0.341 | 0.156 | 0.540 | -0.249 | 1.819 | -13.68 |
| Y217 | 0.695 | 0.623 | 0.491 | 0.565 | -0.106 | 2.167 | -4.89 |
| Q218 | 0.960 | 0.242 | 0.910 | 1.287 | -0.169 | 1.819 | -9.27 |
| K219 | 0.783 | 1.369 | 1.388 | 1.587 | 0.412 | 2.049 | 20.10 |
| E220 | 0.943 | 1.263 | 0.638 | 0.947 | -0.310 | 1.771 | -17.53 |
| S221 | 0.752 | 0.054 | 0.702 | 0.812 | 0.354 | 1.159 | 30.57 |
| Q222 | 0.704 | 0.338 | 0.906 | 0.922 | 0.393 | 1.819 | 21.61 |
| A223 | 0.706 | 0.817 | 0.537 | 0.275 | -0.356 | 1.085 | -32.80 |
| Y224 | 1.565 | 1.095 | 1.222 | 0.934 | -0.252 | 2.167 | -11.63 |
| Y225 | 0.766 | 0.573 | 1.959 | 1.502 | 1.061 | 2.167 | 48.97 |
| D226 | 1.061 | 1.574 | 1.592 | 1.141 | 0.049 | 1.462 | 3.36 |

(^a^) Positive differences in average per-residue SASA indicate a greater solvent exposure in simulations for HRdup in comparison with control M128V simulations.

(^b^) Solvent-accessible surface areas from tri-peptide extended reference conformations (11) were employed as approximate estimates of maximal per-residue SASA.

(^c^) Differences in weighted average per-residue SASA were obtained as percentages of the maximal SASA from (^b^).
