# Supplementary material for: A novel Gerstmann-Sträussler-Scheinker disease mutation defines a precursor for amyloidogenic 8 kDa PrP fragments and reveals N-terminal structural changes shared by other GSS alleles
Source: PLoS Pathog. 2018 Jan 16;14(1):e1006826. doi: 10.1371/journal.ppat.1006826 (PMC5786331; doi:10.1371/journal.ppat.1006826)
Supplement: S6 Table — (DOCX) [file ppat.1006826.s019.docx]

**Supplementary Table S6: Total surface-accessible surface area (SASA) for two trajectories of M128V and HRdup PrP alleles**

Data are calculated from the last 2 ns of the four MD trajectories, and per-residue SASA for models M128V and HRdup in average for two trajectories each.

| **Residue Type** | **Total SASA, nm^2^** | | | |
| --- | --- | --- | --- | --- |
|  | **M128V-I** | **M128V-II** | **HRdup-I** | **HRdup-II** |
| **Hydrophobic residues** | 20.3±3.2 | 19.1±3.1 | 21.3±3.6 | 21.4±3.5 |
| **Charged residues** | 32.9±3.9 | 31.9±3.4 | 30.6±3.6 | 32.1±3.7 |
| **Other residues** | 47.6±7.2 | 44.0±6.2 | 49.0±7.5 | 50.7±7.3 |
| **All residues** | 100.8±14.4 | 95.0±12.7 | 101.0±14.7 | 104.2±14.5 |
|  | **Average per-residue SASA, nm^2^** | | | |
|  | **M128V** | | **HRdup** | |
| **Hydrophobic residues** | 0.548±0.088 | | 0.547±0.091 | |
| **Charged residues** | 1.012±0.115 | | 0.980±0.114 | |
| **Other residues** | 0.655±0.096 | | 0.665±0.098 | |
| **All residues** | 0.709±0.098 | | 0.703±0.100 | |
