# Supplementary material for: A novel Gerstmann-Sträussler-Scheinker disease mutation defines a precursor for amyloidogenic 8 kDa PrP fragments and reveals N-terminal structural changes shared by other GSS alleles
Source: PLoS Pathog. 2018 Jan 16;14(1):e1006826. doi: 10.1371/journal.ppat.1006826 (PMC5786331; doi:10.1371/journal.ppat.1006826)
Supplement: S7 Table — (DOCX) [file ppat.1006826.s020.docx]

**Supplementary Table S7: Residue pairs with robust inter-correlations for Cα atoms indicate PrP regions of the strongest dynamic correlations**

| **PrP allele** | **S1-S2** | **S2-H2** | **H2-H3** | **S2-H3** | **N termini-insert** |
| --- | --- | --- | --- | --- | --- |
| **M128V-I** | L129.Y161 | Y162.C178 | V179.V209 | V160.C213 |  |
|  | G130.V160 | Y161.T182 | C178.C213 | V160.M212 |  |
|  | V128.Y161 | V160.T182 | V179.E210 | V160.V209 |  |
|  | L129.V160 | Y161.C178 | V175.C213 | N158.M212 |  |
|  | G130.Q159 | V160.C178 | V179.C213 |  |  |
|  | Y127.R163 | Y161.I181 | V175.E210 |  |  |
|  | Y127.Y162 | V160.V179 | T182.V209 |  |  |
|  | V128.Y162 |  | C178.V209 |  |  |
|  | Y127.Y161 |  | V175.V214 |  |  |
|  | G130.Y161 |  | C178.E210 |  |  |
|  | S131.Q159 |  |  |  |  |
|  | L129.Y162 |  |  |  |  |
|  | V128.R163 |  |  |  |  |
|  | L129.Q159 |  |  |  |  |
| **M128V-II** | L129.Y161 | Y162.C178 | V179.V209 | V160.C213 |  |
|  | Y127.Y162 | Y161.C178 | C178.C213 | N158.M212 |  |
|  | V128.Y161 | Y161.T182 | V179.E210 | V160.V209 |  |
|  | Y127.R163 | V160.T182 | V175.C213 | V160.M212 |  |
|  | V128.Y162 | V160.C178 | V175.E210 | N158.V209 |  |
|  | Y127.Y161 | Y161.I181 | V179.C213 |  |  |
|  | L129.V160 | V160.V179 | V175.V214 |  |  |
|  | G130.V160 | Y161.V179 | C178.V209 |  |  |
|  | S131.Q159 |  | T182.V209 |  |  |
|  | L129.Y162 |  | C178.E210 |  |  |
|  | G130.Q159 |  |  |  |  |
|  | G130.Y161 |  |  |  |  |
|  | L129.Q159 |  |  |  |  |
|  | S131.N158 |  |  |  |  |
|  | V128.R163 |  |  |  |  |
| **HRdup-I** | L129.Y161 | Y161.T182 | V179.M212 | Y162.C213 | A116.insY7 |
|  | L129.V160 | V160.T182 | V179.C213 | V160.M212 |  |
|  | insV8.Y162 | Y161.I181 | C178.C213 | Y161.C213 |  |
|  | insY7.R163 | Y162.C178 | C178.M212 | Y161.M212 |  |
|  | insY7.Y162 | Y162.T182 | V179.V214 | Y162.M212 |  |
|  | G130.V160 | Y162.I181 |  | V160.C213 |  |
|  | insV8.Y161 | R163.C178 |  |  |  |
|  | insY7.Y161 |  |  |  |  |
|  | insV8.R163 |  |  |  |  |
|  | L129.Y162 |  |  |  |  |
|  | G130.Q159 |  |  |  |  |
|  | G130.Y161 |  |  |  |  |
|  | L129.Q159 |  |  |  |  |
|  | S131.Q159 |  |  |  |  |
| **HRdup-II** | L129.Y161 | Y161.T182 | V179.V209 | Y156.M205 | 116.insG5 |
|  | insY7.Y162 | V160.T182 | N180.V209 |  |  |
|  | insV8.Y161 | Y162.V179 | C178.C213 |  |  |
|  | insV8.Y162 | Y161.V179 | V179.C213 |  |  |
|  | insY7.R163 | Y161.I181 |  |  |  |
|  | insY7.Y161 | Y162.I181 |  |  |  |
|  | L129.V160 | Y162.T182 |  |  |  |
|  | G130.V160 | Y162.C178 |  |  |  |
|  | L129.Y162 | V160.I181 |  |  |  |
|  | insV8.R163 |  |  |  |  |
|  | G130.Y161 |  |  |  |  |
