# Supplementary material for: A novel Gerstmann-Sträussler-Scheinker disease mutation defines a precursor for amyloidogenic 8 kDa PrP fragments and reveals N-terminal structural changes shared by other GSS alleles
Source: PLoS Pathog. 2018 Jan 16;14(1):e1006826. doi: 10.1371/journal.ppat.1006826 (PMC5786331; doi:10.1371/journal.ppat.1006826)
Supplement: S8 Table — (DOCX) [file ppat.1006826.s021.docx]

**Supplementary Table S8: Residues in M128V and HRdup monomers predicted to be involved in dimer contacts**

Data is based on the consensus of top 10 models from three methods (InterEVScore, SOAP_PP, and FRODOCK) with the InterEVDock. Residues highlighted in red are only involved in HRdup contacts.

| M128V-I homodimer | | M128V-II homodimer | | HRdup-I homodimer | | HRdup-II homodimer | |
| --- | --- | --- | --- | --- | --- | --- | --- |
| L124 | L124 | Y127 | Y127 | W98 | K100 | R135 | Q159 |
| Y127 | Y127 | V128 | V128 | K100 | Y148 | T192 | T191 |
| H176 | G123 | P164 | P164 | W144 | W144 | Q159 | Y225 |
| N180 | G125 | S131 | L124 | Y148 | A115 | Y225 | L129 |
| Y168 | P164 | M133 | G125 | N196 | A114 | D226 | G125 |
| M128V-I Hrdup-I heterodimer | | M128V-I HRdup-I heterodimer | | M128V-I Hrdup-II heterodimer | | M128V-II HRdup-I heterodimer | |
| L124 | T200 | M133 | V165 | Y127 | W98 | R135 | Y225 |
| G126 | N196 | Q222 | insV8 | Q167 | W144 | M133 | V111 |
| G125 | K203 | A132 | G125 | G126 | E151 | P136 | N196 |
| Y127 | K100 | N158 | P164 | K184 | Q159 | Y154 | L124 |
| P164 | W144 | P157 | Y225 | R163 | Y148 | A116 | E220 |
